# Integrated Electro-Optic Frequency Combs: Physical Mechanisms, Device Architectures, Material Platforms and System Applications

**DOI:** 10.3390/nano16090559

**Published:** 2026-05-01

**Authors:** Hanqing Zeng, Qingyuan Hu, Yuebin Zhang, Xin Liu, Yongyong Zhuang, Zhihong Wang, Xiaoyong Wei, Zhuo Xu

**Affiliations:** Electronic Materials Research Laboratory, Key Laboratory of the Ministry of Education & International Center for Dielectric Research, School of Electronic Science and Engineering, Xi’an Jiaotong University, Xi’an 710049, China; hqzeng5@gmail.com (H.Z.); zybzgxx@163.com (Y.Z.); eudoraliu@xjtu.edu.cn (X.L.); zhuangzhuang235@xjtu.edu.cn (Y.Z.); zhihong.wang@xjtu.edu.cn (Z.W.); xuzhuo@mail.xjtu.edu.cn (Z.X.)

**Keywords:** electro-optic effect, integrated photonics, material platforms, optical frequency combs

## Abstract

Electro-optic frequency combs (EOFCs), generated through the microwave-driven modulation of continuous-wave lasers, have emerged as a highly reconfigurable and system-compatible class of optical frequency combs with growing importance in microwave photonics, coherent communications, spectroscopy, and precision metrology. In contrast to mode-locked lasers and Kerr microresonator combs, EOFCs offer electrically programmable repetition rates, deterministic phase coherence, and intrinsic compatibility with radiofrequency electronic systems, making them particularly attractive for integrated and application-oriented implementations. As EOFCs evolve toward broader bandwidths, lower power consumption, and full on-chip integration, their achievable performance is increasingly constrained by the interplay between electro-optic physical mechanisms, modulator architectures, and material platform properties. This review establishes a unified analytical framework that systematically connects EOFC generation mechanisms, device configurations, key performance metrics, and platform-level limitations. We first summarize the fundamental electro-optic effects underpinning EOFC generation and analytically examine representative modulator architectures, including phase modulators, Mach–Zehnder modulators, and microresonator-based schemes, to clarify their respective comb-generation characteristics. Key performance determinants, such as modulation depth, bandwidth, electro-optic efficiency, and optical loss, are then discussed to elucidate their coupled influence on comb-line count, spectral flatness, output power, and phase noise. Subsequently, the performance of EOFCs implemented on major integrated platforms, including Silicon on Insulator (SOI), Indium Phosphide on Insulator (InPOI), Lithium Niobate on Insulator (LNOI), and Lithium Tantalate on Insulator (LTOI), is comparatively reviewed to highlight the material-dependent advantages and constraints. Finally, emerging directions based on heterogeneous integration and ferroelectric materials with ultrahigh electro-optic coefficients are discussed as promising pathways to overcome the current performance bottlenecks. This review provides clear physical insights and engineering guidance for the future development of high-performance, integrated EOFC systems.

## 1. Introduction

An optical frequency comb (OFC) is a spectral structure consisting of a series of mutually coherent frequency components with uniform spacing, which corresponds to a train of evenly spaced ultrashort pulses in the time domain [1,2,3,4]. Following the development of the first mode-locked laser (MLL) by Hargrove et al. in 1964 [5] and the initial experimental observation of an optical frequency comb, this field has experienced rapid progress, particularly within laser spectroscopy. By the late 1990s, high-precision optical frequency comb synthesizers were realized, followed by the implementation of octave-spanning f–2f self-referenced combs that enabled simultaneous stabilization of the repetition rate f_r_ and carrier–envelope offset frequency f_0,_ thereby establishing a coherent link between the optical and radio frequency (RF) domains. These landmark achievements culminated in the 2005 Nobel Prize in Physics awarded to Hall and Hänsch, subsequently catalyzing the emergence of new functionalities and transformative technologies across the electromagnetic (EM) spectrum [5,6,7].

**Figure 1:**
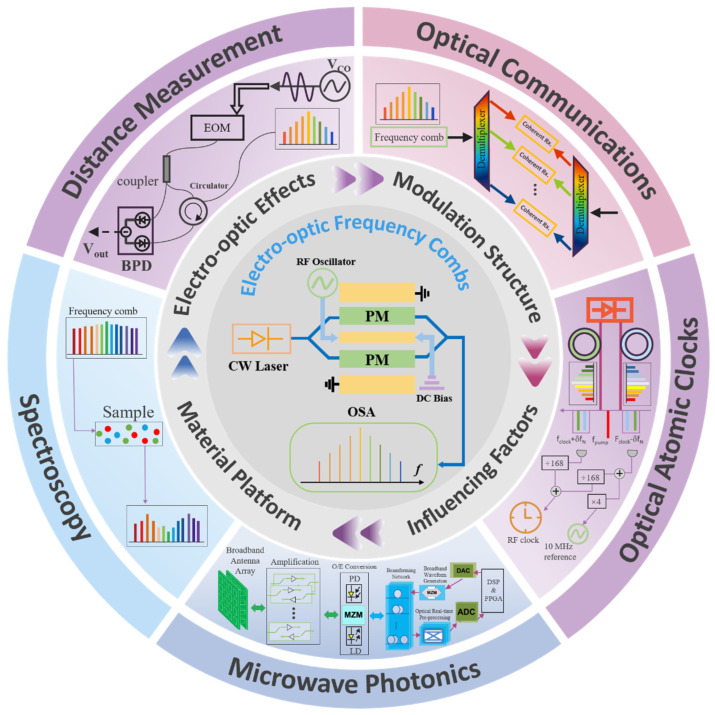
Applications of Integrated EOFCs: distance measurement [33], optical communications [34], optical atomic clocks [35], microwave photonics [36] and spectroscopy [37].

As a natural “ruler” for spectral analysis, optical frequency combs enable absolute optical frequency measurements and coherent optical–microwave interconnection, and have therefore found widespread applications in precision metrology [8,9,10,11,12], optical communications [13,14,15,16], spectroscopy and sensing [17,18,19], and microwave photonics [20,21,22]. At present, OFCs can be broadly classified into three main categories: mode-locked-laser frequency combs (MLLs) [23,24,25,26], Kerr microresonator frequency combs (Kerr combs) [27,28,29,30], and electro-optic frequency combs (EOFCs) [31,32]. In particular, EOFCs, which are generated by electro-optic modulation of a continuous-wave laser under RF driving, offer highly programmable repetition rates and spectral structures, excellent phase stability, and intrinsic compatibility with microwave electronic systems, making them especially attractive for microwave photonics, coherent communication, and precision metrology. A holistic overview of the role of EOFCs in bridging device physics, system architectures, and application scenarios is presented in Figure 1.

MLL-based frequency combs rely on intracavity mode-locking to synchronize the phases of longitudinal modes, producing highly coherent ultrashort pulse trains in the time domain. Consequently, superior spectral purity and low-phase noise can be realized in MLL-based frequency combs. However, the repetition rate is fundamentally constrained because the cavity length, relatively large footprint, and limited tunability restrict practical flexibility [38,39,40,41,42]. Kerr microresonator frequency combs, in contrast, exploit nonlinear processes such as four-wave mixing in high-Q micro-ring resonators to generate densely spaced comb lines, offering strong potential for chip-scale integration. Nevertheless, Kerr microresonator frequency combs are prone to complex nonlinear dynamical behaviors and impose stringent requirements on pump stability, dispersion engineering, and cavity parameters [26,43,44].

In comparison, electro-optic frequency combs are generated through multi-order sideband formation in electro-optic modulators driven by microwave signals, with comb-line amplitude equalization achieved via control of the modulation index and device nonlinearity. EOFCs not only feature strong reconfigurability, high output power, and excellent operational stability but also allow precise control over the comb spectral shape, flatness, pulse width, and repetition rate. Moreover, by leveraging the amplitude, frequency, and polarization properties of the optical carrier, EOFC spectra can be flexibly tailored to meet specific measurement requirements [21,45,46,47]. Notably, electro-optic modulation architectures are particularly well suited to cascaded implementations: multi-stage modulators enable efficient and scalable expansion of comb bandwidth and line count, whereas seamless integration with other optical effects or devices further enhances performance and extends functionality [48,49]. Owing to their exceptional tunability, engineering compatibility, and scalability in cascaded configurations, electro-optic frequency combs have emerged as a central research focus in the development of integrated and application-oriented optical frequency comb technologies.

As EOFCs continue to evolve toward a broader bandwidth, lower power consumption, higher stability, and full on-chip integration, their achievable performance boundaries are increasingly determined by the underlying material and fabrication platforms [50,51,52,53]. Distinct integrated platforms exhibit fundamental differences in electro-optic coefficients, optical mode confinement, bandwidths, thermal stability, etc. These disparities not only affect the driving voltage, modulation depth, and modulation efficiency of electro-optic modulators, but also critically determine key EOFC metrics, such as the attainable number of comb lines, spectral flatness, power dynamic range, and phase noise [54,55,56]. For example, the lithium-niobate-on-insulator (LNOI) platform enables large modulation indices and ultrahigh microwave bandwidths owing to its strong χ^2^ and ultralow-loss waveguides [57,58,59]. In contrast, the indium phosphide-on-insulator (InPOI) platform offers a unique advantage in the monolithic integration of light sources, modulators, and detectors, making it particularly suitable for high-speed communication-oriented EOFC implementations [60,61,62]. Silicon-on-insulator (SOI) and silicon nitride (SiN) platforms, despite lacking intrinsic second-order nonlinearity, benefit from mature CMOS-compatible fabrication and can be synergistically combined with χ^3^-based nonlinear spectral broadening stages to achieve high repetition rates and broadband comb spectra [63,64]. Consequently, the overall performance of EOFCs is governed not only by modulator architectures and driving schemes but also by the combined constraints imposed by the material properties, process parameters, and platform-level system compatibility. From this perspective, a systematic elaboration of EOFC generation mechanisms, key device configurations, performance-limiting factors, and representative implementations across different material platforms is essential for clarifying application suitability and performance advantages, as well as guiding future design optimization and process development of integrated EOFC systems.

Generally, the performance of EOFCs is jointly determined by their underlying electro-optic physical mechanisms, modulator architectures, and material-platform characteristics. Therefore, developing a unified understanding of the intrinsic relationships between these factors is crucial for elucidating the ultimate performance limits and viable optimization pathways of EOFCs. Nevertheless, much of the existing literature tends to concentrate on individual aspects, such as a particular modulator configuration, specific material platform, or single physical mechanism, without providing an integrated perspective that systematically connects generation mechanisms, modulator architectures, key performance determinants, and platform-specific implementations. Hence, this review begins with the fundamental physical origins of electro-optic modulation and systematically summarizes the principal mechanisms underlying EOFC generation, with an emphasis on the four dominant electro-optic effects that constitute the core modulation pathways. It then introduces and analyzes the structural characteristics of mainstream electro-optic modulators, including phase modulators (PMs), Mach–Zehnder modulators (MZMs), and microresonator modulators (MRMs). Modulation processes were analytically derived to establish a unified theoretical framework for understanding the resulting comb spectral characteristics. In addition, the key factors governing EOFC performance, such as modulation depth, device bandwidth, electro-optic efficiency, and driving power requirements, are discussed to clarify the multidimensional optimization landscape of EOFCs. Finally, the review compares the performance of representative integrated material platforms, including SOI, InPOI, and LNOI, within the EOFC context and systematically summarizes their respective advantages and limitations in terms of modulation efficiency, bandwidth scalability, power handling capability, integration density, and process compatibility. Through the construction of this comprehensive analytical framework, this review provides clear engineering guidance and valuable reference for future technological evolution and application development of electro-optic frequency combs.

## 2. The Basic Physical Effects and Modulation Pathway of Electro-Optic Modulation

**Figure 2:**
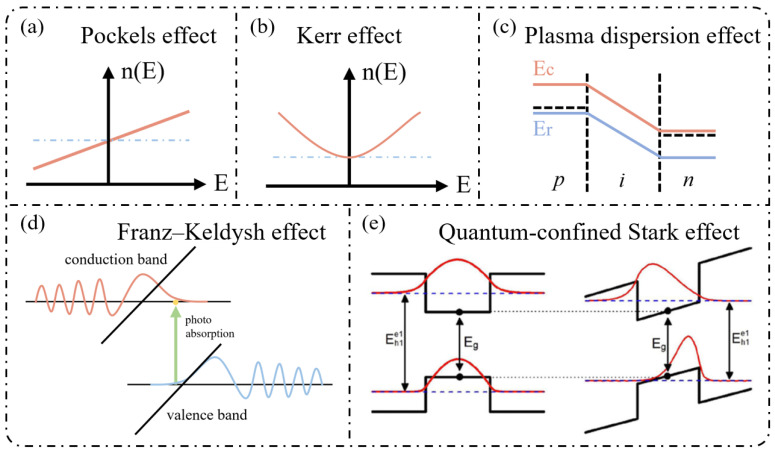
The basic physical effect of electro-optic modulation [67,68,69,70,71,72]: (**a**) Pockels effect; (**b**) Kerr effect; (**c**) plasma dispersion effect; (**d**) Franz–Keldysh effect; (**e**) quantum-confined Stark effect.

Electro-optic modulation enables dynamic control of optical phase, amplitude, and frequency through an externally applied electric field that modifies the refractive index or absorption properties of a material [65,66]. Although the implementation varies across material platforms and device architectures, the underlying mechanisms can be broadly categorized into four fundamental effects: the linear electro-optic (Pockels) effect, the quadratic electro-optic (Kerr) effect, the plasma-dispersion effect, and the electro-absorption effect (Figure 2) [67,68,69,70,71,72].

These mechanisms define the achievable modulation efficiency, bandwidth, and loss, and therefore, directly determine the performance limits of electro-optic frequency comb generation across different platforms.

### 2.1. Linear Electro-Optic (Pockels) Effect

The Pockels effect is one of the most fundamental and widely employed physical mechanisms to achieve high-speed electro-optic modulation. Its essence lies in the linear variation in the refractive index induced by an externally applied static or microwave electric field in non-centrosymmetric media, which in turn enables controllable modulation of the optical phase of the propagating light [73]. When an electric field E is applied to the material, the resulting perturbation of the refractive index tensor can be expressed as follows [74]:

(1) $$∆n\approx -\frac{1}{2}n^{3}r_{ij}E_{j}$$

where n denotes the intrinsic refractive index of the material, r_ij_ is the electro-optic coefficient, which quantifies the response strength of the refractive index (or dielectric tensor) variation in the *i*-th optical component induced by an externally applied electric field along the *j*-th crystallographic direction, and E_j_ represents the j-th component of the applied electric field. The Pockels effect exists predominantly in materials with non-centrosymmetric crystal structures, such as LiNbO_3_, BaTiO_3_, GaAs, and certain KTP-family crystals [51,75]. Owing to its ultrafast intrinsic response (with a fundamental bandwidth approaching the terahertz regime), low insertion loss, high linearity, and high modulation efficiency, the Pockels effect is particularly well suited for Mach–Zehnder modulators on thin-film lithium niobate platforms and high-performance EOFC generation [76]. Despite their superior modulation characteristics, Pockels-effect-based electro-optic modulators also face challenges related to material processing complexity, relatively large device footprints, and limited compatibility with standard CMOS fabrication processes. In addition, electro-optic coefficients exhibit strong crystallographic anisotropy, making precise material orientation and careful device design essential. These factors collectively constrain the widespread adoption of Pockels-based modulators in large-scale CMOS-integrated photonic systems.

### 2.2. Quadratic Electro-Optic (Kerr) Effect

The Kerr effect is a universal refractive-index-based electro-optic modulation mechanism that exists in all dielectric materials. Its physical origin lies in the second-order response of material polarization to an externally applied electric field [30,77]. Under a static or low-frequency electric field, the induced change in the refractive index of the medium can be expressed as

(2) $$∆n=\frac{1}{2}n_{0}^{3}kE^{2}$$

where n_0_ is the intrinsic refractive index, k is the Kerr constant, and E is the electric field strength. The Kerr effect is ubiquitous in centrosymmetric media, including silicon, glass, and silicon nitride, and reflects the second-order nonlinear refractive index response of a material under a strong electric field. Because this effect does not rely on non-centrosymmetric crystal structures, it exhibits broad material universality and excellent compatibility with CMOS photonic platforms [78,79,80]. However, it is generally not regarded as a primary electro-optic modulation mechanism.

Owing to the relatively small Kerr coefficients of most materials, the achievable refractive index modulation based on the Kerr effect is typically weak under practical electric-field strengths. Consequently, effective device implementation often relies on resonant enhancement in microcavities, electric-field concentration in slot waveguides, or ultrahigh-Q resonant structures to substantially amplify the effective phase modulation depth. Meanwhile, excessively high electric fields may induce dielectric breakdown, whereas strong optical fields can trigger thermo-optic effects and photothermal heating, both of which may adversely affect the spectral stability and phase noise of the generated frequency combs.

### 2.3. Plasma Dispersion Effect

The plasma dispersion effect is one of the core mechanisms enabling high-speed electro-optic modulation in semiconductor photonic devices [81]. Its physical origin lies in the modulation of free-carrier concentrations in a semiconductor material through an externally applied voltage, which in turn induces changes in both the refractive index and absorption coefficient, allowing controllable modulation of the optical phase or amplitude [82]. In silicon-based devices, the resulting refractive-index variation can be approximately described as follows [66,83]:

(3) ∆n∝∆N

where ΔN denotes the change in the free-carrier concentration. The plasma dispersion effect is predominantly observed in silicon and III–V semiconductors, where carrier injection or depletion is realized through p-n or p-i-n junction structures. A key advantage of this effect is its ability to achieve high-speed optical modulation within a compact device footprint, with response bandwidths reaching tens of gigahertz. In addition, its excellent compatibility with mature CMOS fabrication processes makes it particularly suitable for large-scale on-chip photonic integrations [81].

In silicon-based Mach–Zehnder modulators and ring modulators, the plasma-dispersion effect enables efficient phase control of propagating light, facilitating broadband EOFC generation and compact device implementation, thereby providing a technological foundation for miniaturized and highly integrated photonic systems. However, plasma-dispersion-based modulation has intrinsic limitations. The achievable refractive-index modulation is relatively modest, and carrier injection or depletion is inevitably accompanied by additional optical absorption, which introduces insertion loss and degrades the overall modulation efficiency as well as the high-power handling capability [84,85]. Moreover, device performance is highly sensitive to carrier-profile engineering and doping uniformity, necessitating careful trade-offs between modulation bandwidth, modulation depth, and optical loss in practical implementations.

### 2.4. Electro-Absorption Effect (Franz–Keldysh/Quantum-Confined Stark Effect)

The electro-absorption effect is an important mechanism for optical amplitude modulation, in which an externally applied electric field modifies the optical absorption properties of a semiconductor material. In bulk semiconductors, the Franz–Keldysh (FK) effect leads to enhanced absorption near the band edge under an applied electric field, whereas in quantum-well structures, the quantum-confined Stark effect (QCSE) induces spatial separation of the electron and hole wavefunctions in the presence of an electric field, resulting in a redshift of the excitonic absorption peak and enabling efficient electro-optic modulation [67,68,86,87].

The primary advantage of electro-absorption is its ability to achieve strong intensity modulation within a compact device footprint, offering a large modulation depth and high-speed response that can extend from tens to even hundreds of gigahertz. These characteristics make electro-absorption modulation particularly attractive for high-speed optical communications and densely integrated photonic systems [88,89]. In quantum-well-based devices, the electric-field-induced separation of electron–hole wavefunctions enables precise control of the absorption spectrum, providing an effective route toward compact electro-optic modulators and high-speed EOFC implementations.

Nevertheless, electro-absorption modulation has several inherent limitations. Strong electric fields tend to introduce significant optical loss, while stringent requirements on quantum-well design and fabrication precision increase the process complexity. In addition, achieving a high bandwidth and modulation efficiency must be carefully balanced against power-handling capability and thermal management considerations. As a result, practical device designs must strike a trade-off between the modulation performance and long-term reliability.

Overall, these electro-optic mechanisms present distinct trade-offs in terms of modulation efficiency, bandwidth, loss, and integration compatibility, which fundamentally constrain the achievable EOFC performance across different material platforms.

## 3. Typical Electro-Optic Modulator Structure and Comb Spectrum Derivation

**Figure 3:**
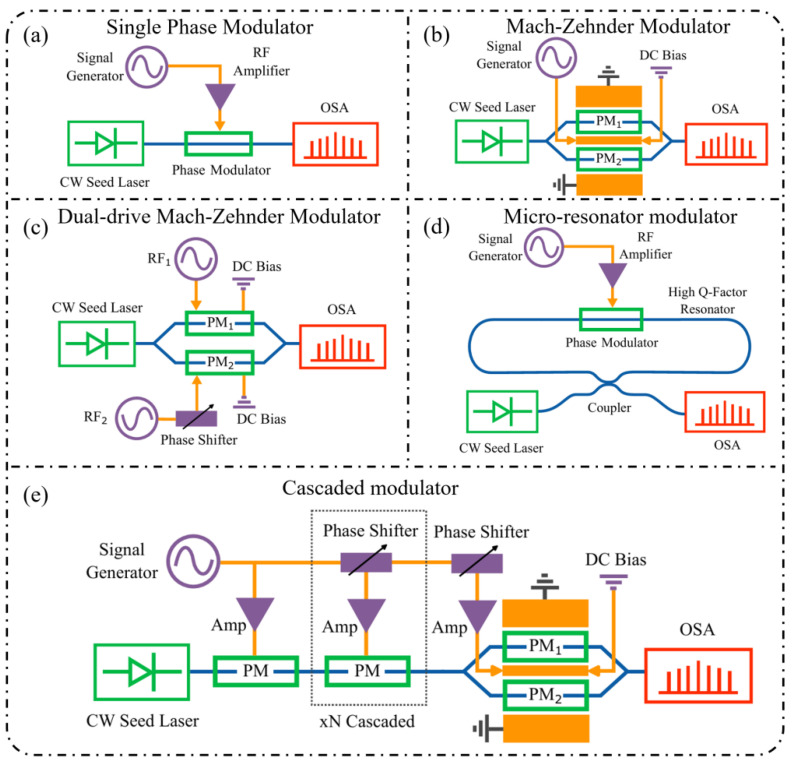
Various schematics of different EO comb generator architectures [90,91,92,93]: (**a**) PM; (**b**) MZM; (**c**) DDMZM; (**d**) MRM; and (**e**) cascaded modulator.

The realization of electro-optic frequency combs (EOFCs) relies not only on the underlying electro-optic mechanisms but also on the design of modulator architectures that translate electrical driving signals into controlled optical phase and amplitude modulation, enabling multi-order sideband generation. From a performance perspective, key parameters such as modulation depth, bandwidth, insertion loss, and optical–microwave overlap collectively determine the achievable comb line number, spectral flatness, and scalability. As shown in Figure 3, the main electro-optic modulator architectures include phase modulators (PMs), Mach–Zehnder modulators (MZMs), dual-drive MZMs (DDMZMs), and micro-ring modulators (MRMs) [90,91,92,93].

These architectures represent distinct modulation pathways, offering different trade-offs between efficiency, spectral control, bandwidth scalability, and system complexity, which are critical for EOFC design and optimization.

### 3.1. Phase Modulator (PM)

When a continuous-wave optical field $E_{in}(t)=E_{0}e^{i\omega_{0}t}$ propagates through a single-stage PM based on the linear electro-optic (Pockels) effect, plasma dispersion effect, or Kerr effect, the optical phase is periodically modulated by an externally applied microwave signal. The resulting output optical field can be written as follows [90,94,95,96]:

(4) $$E_{out}(t)=E_{0}e^{i\omega_{0}t+i\beta cos(\Omega t)}$$

where β denotes the phase-modulation index, and Ω is the angular frequency of the microwave driving signal. By applying the Jacobi–Anger expansion,

(5) $$e^{i\beta cos(\Omega t)}=\sum_{n=-\infty }^{+\infty }i^{n}J_{n}(\beta )e^{in\Omega t}$$

the output optical field can be expressed in the frequency domain as follows:

(6) $$E_{out}(t)=E_{0}\sum_{n=-\infty }^{+\infty }i^{n}J_{n}(\beta )e^{i(\omega_{0}+n\Omega )t}$$

This formulation clearly indicates that under sinusoidal RF driving, a PM generates a series of equally spaced high-order modulation sidebands symmetrically distributed around the optical carrier frequency. The frequency spacing between adjacent sidebands is given by the modulation angular frequency Ω, whereas the amplitude of each sideband is determined by the Bessel function Jn(β), which depends explicitly on the modulation index β.

### 3.2. Mach–Zehnder Modulator (MZM)

As shown in Figure 3b, as a representative interferometric modulation architecture, the MZM converts electro-optic phase modulation into optical intensity (amplitude) modulation by introducing controlled phase shifts in its two interferometer arms such that the recombined optical fields interfere in a tunable manner. The output optical field of an MZM can be expressed as follows [97,98,99]:

(7) $$E_{out}(t)=\frac{E_{0}}{2}\left[e^{i\phi_{1}(t)}+e^{i\phi_{2}(t)}\right]e^{i\omega_{0}t}$$

We assume that one arm is driven by a modulation signal, while the other arm is subject only to a static bias phase ϕ_b_, defined as follows:

(8) $$\phi_{1}\left(t\right)=\phi_{b}+\beta \cos\left(\Omega t\right),\;\;\phi_{2}\left(t\right)=0$$

and the output field becomes the following:

(9) Eout(t)=E0ei(ω0t+ϕb2)cosϕb+βcosΩt2

This expression reveals that the MZM transfer function inherently contains both phase and amplitude modulation components. Consequently, under asymmetric DC biasing conditions, effective amplitude–phase coupling is introduced, allowing both odd- and even-order modulation sidebands to coexist in the output spectrum.

### 3.3. Dual-Drive MZM (DDMZM)

Compared with a conventional MZM, the dual-drive configuration, schematically presented in Figure 3c, permits independent RF excitation of the two interferometer arms, thereby introducing a controllable relative phase difference. As a result, the recombined optical field exhibits joint amplitude and phase modulation, offering a substantially higher degree of freedom in spectral control than single-drive MZMs. When equal-amplitude but opposite-sign phase modulation is applied to the two arms, the phase shifts can be written as [100,101]:

(10) $$\phi_{1,2}\left(t\right)=\phi_{b}\pm \beta \cos\left(\Omega t\right)$$

The output optical field is then given by the following:

(11) $$E_{out}(t)=\frac{E_{0}}{2}\left[e^{i\left[\phi_{b}+\beta \cos\left(\Omega t\right)\right]}+e^{i\left[\phi_{b}-\beta \cos\left(\Omega t\right)\right]}\right]e^{i\omega_{0}t}$$

By simplification, this expression reduces to the following:

(12) $$E_{out}(t)=E_{0}e^{i{(\omega }_{0}t+\phi_{b})}cos\left[\beta \cos\left(\Omega t\right)\right]$$

This result indicates that by independently driving the two arms and properly adjusting the bias point, the DDMZM can exploit interferometric control to selectively suppress the optical carrier and even-order sidebands, enabling comb spectral flattening or single-sideband enhancement. Because of this capability, DDMZMs play a pivotal role in high-performance EOFC generation and single-sideband modulation schemes.

### 3.4. Micro-Ring Modulator (MRM)

The MRM exploits resonant cavity enhancement to strengthen the optical–electrical interaction, enabling enhanced phase modulation or amplitude–phase coupling, as shown in Figure 3d. For the same input continuous-wave optical field $E_{in}(t)=E_{0}e^{i\omega_{0}t}$, the single-stage transmission function of a micro-ring resonator can be expressed as follows [102,103,104,105,106]:

(13) $$E_{out}(t)=E_{in}(t)\frac{t-ae^{i\left[\phi +\beta \cos\left(\Omega t\right)\right]}}{1-ate^{i\left[\phi +\beta \cos\left(\Omega t\right)\right]}}$$

where t denotes the cavity–waveguide coupling coefficient, a is the intracavity loss factor, ϕ represents the static detuning phase, β is the phase-modulation index, and Ω is the modulation angular frequency. Similar to the DDMZM case, the optical field in a micro-ring resonator undergoes multiple round trips, leading to intracavity accumulation of the phase modulation and, consequently, a significant enhancement of the higher-order sideband amplitudes.

When the modulation index satisfies β ≪ 1, the phase modulation term can be approximated using small-signal expansion:

(14) $$e^{i\beta \cos\left(\Omega t\right)}\approx 1+i\beta \cos\left(\Omega t\right)\approx 1+\frac{i\beta }{2}\left(e^{i\Omega t}+e^{-i\Omega t}\right).$$

Substituting this approximation into the cavity transmission function and expanding the result yields an approximate frequency-domain representation of the output field:

(15) $$E_{out}(t)\approx E_{0}e^{i\omega_{0}t}\sum_{n=-\infty }^{+\infty }C_{n}(\phi ,t,a,\beta )e^{in\Omega t}$$

where the coefficient $C_{n}(\phi ,t,a,\beta )$ denotes the complex amplitude of the nth-order sideband after cavity enhancement. Its explicit form is jointly determined by the cavity-coupling condition, intrinsic loss, and static detuning. In MRMs, high-order sidebands are significantly amplified through multiple round-trip interferences within the resonator, enabling the formation of dense and spectrally flattened combs. This enhancement can be approximately expressed as

(16) $$C_{n}\sim \sum_{m=0}^{\infty }{\left(ate^{i\phi }\right)}^{m}J_{n}(\beta )$$

where the Bessel function Jn(β) describes the sideband generated by a single-phase modulation event, and the geometric series $\sum_{m=0}^{\infty }{\left(ate^{i\phi }\right)}^{m}$ represents the cumulative cavity-enhancement factor arising from repeated round trips. Analogous to DDMZMs, the sideband distribution in MRMs can be engineered by adjusting the static detuning phase ϕ, coupling coefficient t, and cavity loss factor a, allowing the selective control of the sideband gain for spectral flattening, carrier suppression, or targeted sideband enhancement. Benefiting from resonant enhancement, MRMs enable the generation of high-density phase-coherent optical frequency combs within a compact footprint, making them particularly attractive for integrated on-chip EOFC systems [107,108].

### 3.5. Cascaded Modulation

In EOFC systems, cascaded modulation schemes are widely adopted to further extend the comb bandwidth and increase the number of comb lines. Typical implementations, as illustrated in Figure 3e, include serially cascading multiple PMs or combining PMs with other modulator types, such as MZMs, DDMZMs, or MRMs. As an illustrative example, we consider a two-stage cascaded PM-PM configuration [109,110,111]. For an input continuous-wave optical field $E_{in}\left(t\right)=E_{0}e^{i\omega_{0}t}$, the first-stage PM, driven at angular frequency Ω with modulation index β_1_, produces an output field given by the following:

(17) $$E_{1}\left(t\right)=E_{0}e^{i\omega_{0}t}e^{i\beta_{1}\cos\left(\Omega t\right)}=E_{0}e^{i\omega_{0}t}\sum_{n=-\infty }^{+\infty }i^{n}J_{n}(\beta_{1})e^{in\Omega t}$$

When a second PM with modulation index β_2_ is cascaded and acts on the output of the first stage, the total output field becomes the following:

(18) $$E_{2}\left(t\right)=E_{1}\left(t\right)e^{i\beta_{2}\cos\left(\Omega t\right)}=E_{0}e^{i\omega_{0}t}\sum_{n=-\infty }^{+\infty }i^{n}J_{n}(\beta_{1})e^{in\Omega t}\sum_{m=-\infty }^{+\infty }i^{m}J_{m}(\beta_{2})e^{im\Omega t}$$

By combining the exponential terms, the total output field can be rewritten as follows:

(19) $$E_{2}\left(t\right)=E_{0}e^{i\omega_{0}t}\sum_{k=-\infty }^{+\infty }\left[\sum_{n=-\infty }^{+\infty }i^{k}J_{n}(\beta_{1})J_{k-n}(\beta_{2})\right]e^{ik\Omega t}$$

This expression clearly reveals the sideband accumulation rule in cascaded PM configurations, where the amplitude of the kth-order sideband is given by the convolution sum of the Bessel coefficients associated with each modulation stage. Consequently, the strength of high-order sidebands can be precisely engineered by independently tuning the modulation indices β_1_, β_2_, …, thereby enabling controlled spectral broadening and improved spectral flatness.

Overall, different modulator architectures exhibit intrinsic trade-offs. Phase modulators provide structural simplicity but limited spectral control. Interferometric modulators, such as MZMs and DDMZMs, enable improved spectral shaping at the expense of increased system complexity and bias sensitivity. Resonant modulators offer enhanced modulation efficiency and compact footprints but are constrained by bandwidth and thermal stability.

These competing considerations not only affect device-level performance but also influence system-level design considerations, including RF driving complexity, bias control stability, and scalability of multi-stage integration. Therefore, the selection of modulator architecture depends on the targeted balance between comb bandwidth, spectral flatness, and implementation complexity.

## 4. Key Performance Factors of EOFC

After clarifying the structural characteristics of different electro-optic modulators and the corresponding mechanisms underlying their comb-spectrum formation, it is necessary to further examine the key metrics and limiting factors of EOFCs from a system performance perspective. Although phase modulation, interferometric modulation, and cavity-enhancement mechanisms can support multi-order sideband generation and spectral broadening from narrowband to ultrabroadband regimes, the ultimate achievable comb performance remains jointly constrained by device-level parameters such as modulation depth, driving bandwidth, optical loss, and microwave-to-optical matching efficiency.

**Figure 4:**
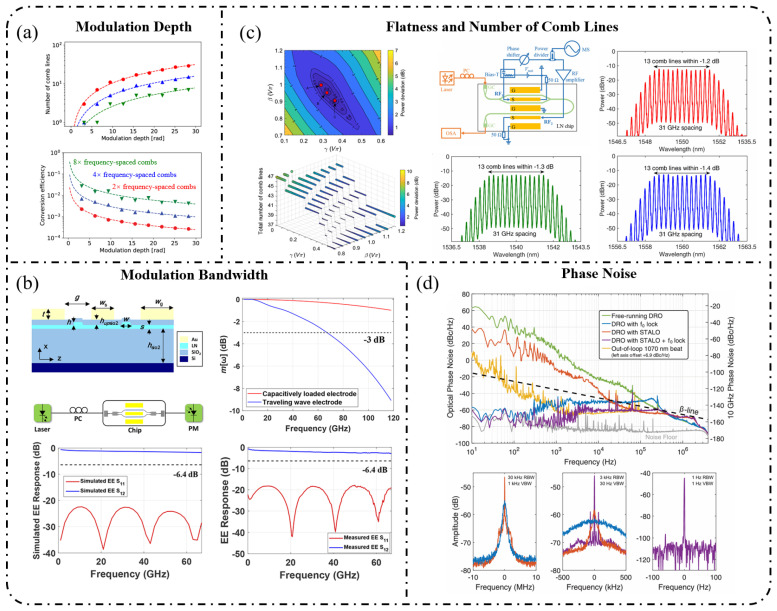
(**a**) Number of comb lines and conversion efficiency versus modulation depth [112]; (**b**) contour of the OFC flatness, experimental setup for flat OFC generation and measured OFC spectra [113,114]; and (**c**) modulation bandwidth [115]; (**d**) EOM-comb phase noise [25].

In practical applications, metrics such as the comb line number, spectral flatness, repetition rate, and output power collectively determine EOFC usability, whereas phase noise and frequency stability directly govern scalability in metrology, optical communications, and microwave photonic scenarios. Figure 4 provides representative illustrations of these key performance metrics. Accordingly, the following discussion starts from these quantitative performance parameters and systematically addresses their physical origins, their coupling mechanisms with different modulator architectures, and the extent to which the material platform sets the attainable performance ceiling. This unified analytical framework is intended to clarify the practical limitations of EOFCs and provide guidance for subsequent platform selection and optimization.

### 4.1. Modulation Depth (β)

The modulation depth β is the primary parameter that determines the achievable spectral bandwidth and number of comb lines in an EOFC [112,113,116]. Physically, β represents the phase excursion imparted to the optical field by an applied RF signal. It defines the effective support range of the Bessel coefficients Jn(β), and therefore, directly sets the maximum order of the generated sidebands n_max_, leading to an approximately linear relationship between the comb bandwidth and modulation depth:

(20) $$n_{max}\approx \beta$$

Consequently, in performance-driven EOFC design, achieving a large effective modulation depth is one of the most critical objectives for broadband comb generation from a physical standpoint. The modulation depth can be expressed as

(21) $$\beta =\frac{\pi {\;V}_{RF}}{V_{\pi }}$$

where V_RF_ denotes the amplitude of the RF driving voltage applied to the modulator electrodes and V_π_ is the half-wave voltage required to induce a π-phase shift. The value of V_π_ is jointly determined by the intrinsic electro-optic response of the material, optical mode confinement, and overlap efficiency between the optical and microwave fields. As a result, different material platforms exhibit fundamentally different capabilities for achieving large modulation depths. For example, materials based on the Pockels effect (such as thin-film lithium niobate) benefit from strong second-order nonlinearity and large electro-optic coefficients, enabling large modulation indices β at relatively low driving voltages and making them well-suited for broadband EOFC generation. In contrast, silicon-based platforms rely on the plasma dispersion effect, where refractive-index modulation is limited by achievable carrier injection or depletion levels, which results in smaller β values and is often accompanied by additional absorption loss. Media that rely solely on the Kerr effect typically exhibit low single-pass modulation efficiency and therefore require high driving power or resonant enhancement to realize effective phase modulation. Accordingly, the type of electro-optic effect fundamentally constrains the upper limit of attainable modulation depth.

In addition to material properties, modulator architecture plays a crucial role in determining the efficiency of the modulation depth. In a single-stage PM, β scales linearly with the effective interaction length and directly governs sideband generation. In Mach–Zehnder modulators, the interferometric operation introduces amplitude–phase coupling, allowing the sideband distribution to be reconfigured through bias-point control. In comparison, in microring modulators, phase modulation accumulates over multiple cavity round-trip, and the effective modulation depth can be expressed as β_eff_ = Fβ (where F is the cavity enhancement factor). This mechanism enables efficient generation of higher-order sidebands within a compact footprint.

From an integrated platform perspective, the LNOI platform benefits from intrinsically large linear electro-optic coefficients and low-loss traveling-wave modulation, allowing a relatively large β to be achieved. The SOI platform, which is constrained by the efficiency of the plasma dispersion effect and its associated absorption loss, typically supports more limited modulation depths. InP-based platforms can enhance the modulation efficiency through electro-absorption or hybrid modulation schemes; however, optical loss and power-handling capability often emerge as the dominant limiting factors.

### 4.2. Modulation Bandwidth

As shown in Figure 4b, the modulation bandwidth characterizes the frequency range over which a modulator can effectively impose optical phase or amplitude modulation under RF driving. It is a critical parameter that determines the highest-frequency sidebands that can be generated in an EOFC, and consequently, the ultimate optical bandwidth that the comb can cover [115,117,118,119]. For phase modulators, the achievable bandwidth is primarily limited by the intrinsic material response time, velocity matching between the optical and microwave fields, and transmission characteristics of the electrodes. In modulators based on the plasma dispersion effect, the modulation speed is governed by the time constants associated with carrier injection or depletion. In contrast, in Pockels-effect-based materials, the intrinsic electro-optic response is nearly instantaneous, and the modulation bandwidth is constrained by extrinsic factors, such as microwave transmission line loss, waveguide capacitance, and impedance matching. An insufficient bandwidth directly suppresses the generation of high-order sidebands, thereby degrading the spectral uniformity and reducing the usable comb bandwidth.

The integrated material platform played a decisive role in setting the upper limit of the achievable modulation bandwidth [120]. On the LNOI platform, the ultrafast response of the Pockels effect, combined with traveling-wave electrode designs and optimized microwave–optical velocity matching, enables effective modulation bandwidths exceeding 50 GHz, making it well-suited for ultrahigh-speed EOFC generation. In contrast, on the SOI platform, plasma-dispersion-based modulation is limited by carrier mobility and injection/depletion rates, with bandwidths typically confined to the tens of gigahertz range, and operation at higher frequencies often leads to sideband roll-off and spectral non-uniformity. The InPOI platform relies on quantum-well electro-absorption for modulation and can achieve high-speed operation; however, under long-wavelength or high-power conditions, thermal effects tend to limit the high-frequency stability.

Overall, the modulation bandwidth is constrained by both the fundamental physical limits of the modulation mechanism and characteristics of the integrated platform. Therefore, it serves as a core performance metric that governs the EOFC-line coverage, spectral broadening rate, and efficiency of high-order sideband generation.

### 4.3. Flatness

Spectral flatness quantifies the uniformity of the power distribution among comb lines within a target bandwidth, and is a key metric for determining the practical usability of EOFCs in applications such as coherent optical communications, microwave photonic links, and multi-channel parallel processing [100,114,121]. An ideal comb spectrum should maintain a nearly uniform power distribution across the operational bandwidth to avoid a signal-to-noise ratio imbalance or reduced channel utilization caused by spectral power fluctuations. From a physical perspective, spectral flatness originates from the energy redistribution among different modulation sidebands during the modulation process and is jointly governed by the form of the modulation function, coupling between phase and amplitude modulation, and generation efficiency of high-order sidebands.

Taking single-stage phase modulation as an example, the amplitude of each sideband is determined by the Bessel function Jn(β), which exhibits oscillatory dependence on the sideband order. Consequently, pronounced power variations were inevitable at finite modulation depths. Consequently, simply increasing the modulation index β is insufficient for achieving a uniformly flat spectral envelope over a wide bandwidth. Instead, additional spectral shaping and energy-redistribution strategies, such as cascaded modulation, spectral filtering, or resonant enhancement, are typically required to realize high-flatness EOFCs [112,113,122].

Different modulator architectures exhibit markedly different capabilities for controlling spectral flatness. Interferometric modulators based on Mach–Zehnder configurations can tailor the weighting of individual comb lines through bias-point selection and driving schemes, whereas differentially driven structures further suppress the carrier or specific sideband orders, thereby improving power uniformity. Architectures incorporating amplitude–phase coupling (e.g., PM-IM cascades or PM-MZM combinations [121,122]) exploit coherent superposition in the frequency domain to compensate for high-order sidebands and are commonly employed to generate flat-top comb spectra. On the other hand, micro-ring modulators (MRMs) leverage intracavity field enhancement to deviate high-order sideband amplitudes from single-pass Bessel decay behavior, enabling improved spectral uniformity under appropriately engineered coupling and detuning conditions. However, achieving high flatness in MRMs often requires a careful trade-off between thermal stability and effective bandwidth.

From an integrated-platform perspective, the LNOI platform, which benefits from large achievable modulation depths and low optical loss, is particularly well-suited for broadband flat-top EOFC generation. In contrast, on the SOI platform, limitations in modulation depth and carrier-induced absorption often necessitate multi-stage or more complex cascaded modulation schemes to improve spectral flatness. The InPOI platform offers strong amplitude-control capability; however, further enhancement of spectral flatness remains constrained by device loss and thermal effects.

### 4.4. Number of Comb Lines and Spectral Expandability

The number of comb lines is a core metric for evaluating the spectral coverage and information-carrying capacity of an electro-optic frequency comb (EOFC). Physically, it reflects the number of resolvable frequency-domain modes and a modulation process can be effectively generated from a continuous-wave optical field [94,123,124]. Unlike spectral flatness, which emphasizes power uniformity, the number of comb lines primarily characterizes the ultimate spectral broadening capability and is jointly determined by the modulation depth and modulation bandwidth. Within the basic phase-modulation model, the output optical field can be expressed as

(22) $$E_{out}(t)=E_{0}\sum_{n=-\infty }^{+\infty }i^{n}J_{n}(\beta )e^{i(\omega_{0}+n\Omega )t}$$

where the effective sideband order is governed by the support range of the Bessel function, which typically satisfies n_max_ ≈ β. For an EOFC with repetition frequency Ω, the number of usable comb lines N can be approximated as follows:

(23) $$N\approx 2n_{max}+1\approx 2\beta +1$$

This relation indicates that under the ideal assumption of a sufficiently large modulation bandwidth, the number of comb line scales approximately linearly with the modulation depth. In practical devices, however, the achievable number of comb lines is constrained not only by the modulation depth, but also by the finite modulation bandwidth. When the modulation frequency approaches the electro-optic bandwidth B_EO_ of the device, the higher-order sidebands experience pronounced attenuation, thereby limiting the maximum usable sideband order. This additional constraint can be expressed as follows:

(24) $$n_{max}\leq \frac{B_{EO}}{\Omega }$$

By combining these two limiting conditions, the effective number of usable comb lines in an EOFC can be written as

(25) $$N\approx 2\;min(\beta ,\frac{B_{EO}}{\Omega })+1$$

These relationships clarify that the modulation depth determines whether high-order sidebands can be generated, whereas the modulation bandwidth determines whether these sidebands can be efficiently transmitted and exploited. Consequently, under high-repetition-rate operation, the number of comb lines may be bandwidth-limited, even when a large modulation depth is available, whereas at low repetition rates, the comb-line count is more often constrained by the achievable modulation depth.

### 4.5. Repetition Rate (f_rep_)

The repetition rate, f_rep_, of an electro-optic frequency comb corresponds to the frequency spacing between adjacent comb lines, and is physically determined by the applied RF driving frequency [125,126]:

(26) $$f_{rep}=\frac{\Omega }{2\pi }$$

Unlike mode-locked lasers or Kerr microresonator combs, the repetition rates of which are intrinsically set by the cavity length or free spectral range (FSR), the repetition rate of an EOFC is not constrained by the optical cavity. Instead, it is directly defined by the microwave source, endowing the EOFCs with exceptional flexibility in repetition-rate tunability and access to high repetition frequencies. In principle, f_rep_ can be continuously tuned over a wide range from hundreds of megahertz to tens or even hundreds of gigahertz, thereby accommodating diverse application requirements.

Importantly, the repetition rate is not an independent parameter but is intrinsically coupled to the modulation depth, modulation bandwidth, and number of comb lines. As the repetition rate increased (i.e., as Ω increased), the maximum achievable sideband order on each side of the carrier decreased, which in turn limited the total spectral coverage of the comb. Consequently, EOFCs operating at high repetition rates are typically characterized by sparser but highly stable comb spectra, whereas those operating at lower repetition rates favor denser combs with broader spectral coverage. This fundamental trade-off between the repetition rate and comb line number constitutes a defining feature of EOFC design and distinguishes EOFCs from microresonator-based frequency combs.

Different modulator architectures exhibit markedly different capabilities under high-repetition-rate operation [127,128,129]. Single-stage phase modulators are primarily limited by their RF driving capability and electrode bandwidth, with their ultimate repetition-rate ceiling determined by optical–microwave velocity matching and RF transmission loss. Mach–Zehnder modulators and their differentially driven variants face similar challenges at high frequencies, including electrode loss and bias-point stability, although differential driving can partially improve high-frequency modulation efficiency. In contrast, the repetition rate of micro-ring modulators is jointly constrained by the optical response time of the cavity and the matching between the modulation frequency and the cavity FSR. When the modulation frequency Ω approaches or exceeds the cavity linewidth, the modulation efficiency degrades significantly, rendering MRMs more suitable for stable operation near specific repetition rates. For cascaded phase-modulation architectures, although sideband accumulation can enhance the effective modulation depth at high repetition rates, the accompanying accumulation of insertion loss and increased RF chain complexity ultimately limits their scalability.

### 4.6. Phase Noise

**Figure 5:**
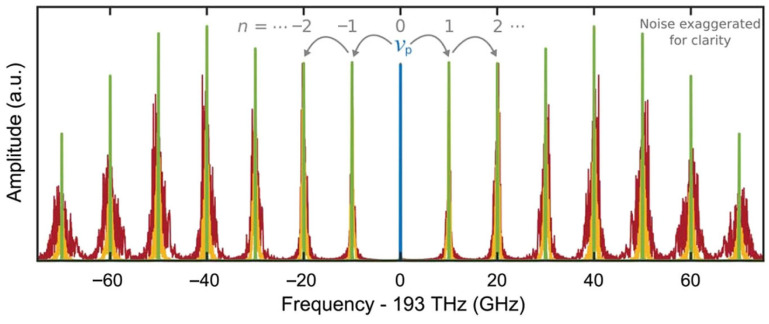
Schematic showing the phase noise accumulation on high-order sidebands of an EOFC (red) compared to a filtered comb (yellow) but also to an ideal stabilized comb (green) [25,130].

Phase noise is a central metric for evaluating the frequency purity and coherence of electro-optic frequency combs (EOFCs), and it directly determines their performance limits in precision metrology, coherent communication, and microwave photonic systems. For EOFCs, the physical origins of phase noise are well defined, and the phase stability of each comb line is primarily inherited from the driving microwave source and optical carrier, whose phase noise is transferred to the comb lines through the electro-optic modulation process, as illustrated in Figure 5 [25,41,130]. For the n-th comb line, the instantaneous angular frequency can be expressed as

(27) $$\omega_{n}=\omega_{0}+n\Omega$$

and the corresponding phase-noise power spectral density approximately follows:

(28) $$S_{\phi ,n}\left(f\right)\approx S_{\phi ,opt}\left(f\right)+n^{2}S_{\phi ,RF}\left(f\right)$$

where $S_{\phi ,opt}\left(f\right)$ and $S_{\phi ,RF}\left(f\right)$ denote the phase-noise contributions from the optical carrier and RF driving source, respectively. This relationship clearly indicates that the EOFC phase noise scales quadratically with the comb-line order, rendering high-order sidebands particularly sensitive to the RF source phase noise. This intrinsic scaling constitutes a fundamental limitation for EOFCs operating in ultra-broadband regimes or relying on high-order comb lines.

From a device-implementation perspective, the phase-noise characteristics of EOFCs are jointly constrained by the modulator architecture and integrated material platform [131,132,133,134]. In single-stage phase modulators, RF phase noise is transferred to the optical sidebands with minimal distortion, representing the most direct phase-noise mapping mechanism. Mach–Zehnder modulators and their differentially driven variants, while offering greater flexibility for spectral shaping, are more sensitive to bias drift and thermal perturbations owing to their interferometric nature, potentially introducing additional low-frequency phase noise. Multi-stage or cascaded modulation architectures can further extend the comb bandwidth; however, the accumulation of RF chain noise may degrade the phase stability of high-order comb lines.

From an integrated platform standpoint, the intrinsically low material noise of the LNOI platform implies that the EOFC phase noise is typically dominated by the stability of the external RF source and the seed laser. In contrast, plasma-dispersion-based modulation on the SOI platform is susceptible to carrier density fluctuations and absorption variations, which can introduce amplitude–phase noise coupling. For InP-based platforms, electro-absorption and quantum-well structures are particularly sensitive to temperature and electric-field fluctuations. Consequently, phase-noise performance under high-frequency or high-power operation requires careful attention to thermal management and device-stability design.

### 4.7. Comb Power

Comb power is a key performance metric that determines the practical usability of electro-optic frequency combs (EOFCs). It typically refers to the optical power carried by the individual comb lines and their distribution across the frequency domain [135,136,137,138]. Unlike the number of comb lines and spectral flatness, comb power directly governs system-level figures of merit, such as the signal-to-noise ratio, dynamic range, and operational margin of subsequent detection or signal-processing stages. In an ideal phase modulation model, the input optical power is redistributed among different modulation sidebands in the frequency domain. The optical power of the n-th comb line can be approximated as

(29) $$P_{n}=P_{0}{\left|J_{n}(\beta )\right|}^{2}$$

where P_0_ is the input optical power, and J_n_(β) is the n-th-order Bessel function. This expression indicates that in the absence of loss, the comb power distribution is determined solely by the modulation depth and obeys energy conservation across the sidebands. However, in practical devices, the insertion loss, finite modulation efficiency, and structure-dependent losses significantly affect both the absolute power level of the comb and its distribution among individual lines.

From a physical standpoint, the comb power is primarily constrained by three factors: the available input optical power, optical losses introduced during the modulation process, and the efficiency of power redistribution among different sideband orders [101,139,140]. As the modulation depth increases, the optical power spreads over a larger number of higher-order sidebands, leading to an inevitable reduction in the average power per comb line. Meanwhile, the waveguide propagation loss, electrode absorption, carrier-induced absorption, and coupling losses further diminish the total output power of the comb. Consequently, while pursuing large comb-line counts and broadband spectral coverage, comb power often emerges as a critical bottleneck for overall system performance, necessitating a careful trade-off between the spectral broadening capability and the available system power budget.

## 5. Performance of EOFCs on Different Integrated Platforms

After systematically reviewing the modulation mechanisms, device architectures, and key performance metrics of EOFCs, it can be further emphasized that the ultimate performance limits of EOFCs are not solely determined by the modulator structures. At a more fundamental level, they are strongly constrained by the integrated material platforms on which they are implemented. Distinct materials exhibit substantial differences in their intrinsic physical properties, such as refractive index, electro-optic coefficients, nonlinear optical response, propagation loss, and process compatibility, which collectively define the attainable ranges of critical performance metrics, including modulation depth, operational bandwidth, power-handling capability, phase noise, and long-term stability.

Accordingly, a systematic comparison of the EOFC performance from the perspective of integrated material platforms is essential for gaining deeper insight into the performance disparities among different implementation routes and for evaluating their respective engineering suitability. To facilitate this analysis, Table 1 summarizes the key material properties of representative integrated photonic platforms for electro-optic frequency comb generation, highlighting the fundamental parameters that govern modulation efficiency, bandwidth scalability, loss characteristics, and integration compatibility. Such a structured comparison provides a clear physical basis for understanding how material-level constraints map onto system-level EOFC performance.

### 5.1. EOFC on the SOI

To overcome the intrinsic trade-offs among modulation efficiency, bandwidth, and spectral flatness, recent EOFC implementations on the SOI platform have progressively converged toward three physically unified architectural routes: single-stage modulation, aimed at compact and low-complexity comb sources; cascaded modulation, designed to achieve stronger spectral shaping capability and a larger usable comb bandwidth; and resonant-enhanced modulation, which exploits multiple round trips of the optical field in feedback cavities to realize effective multi-stage modulation and field enhancement. Representative experimental realizations of these schemes are comparatively summarized in Table 2.

**Figure 6:**
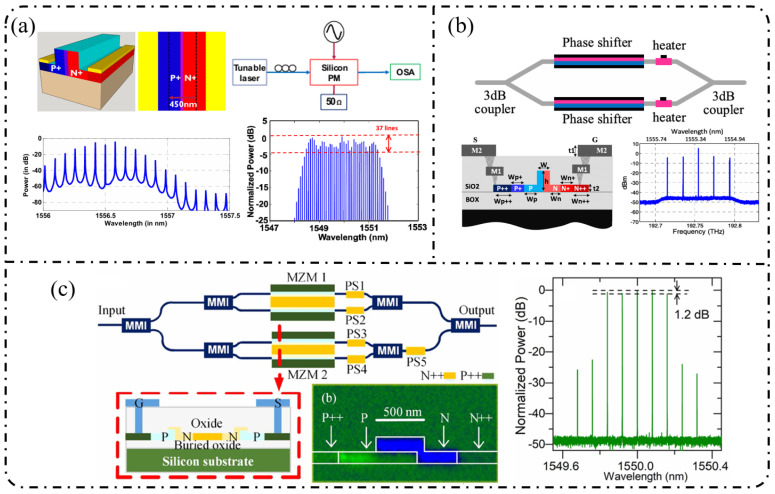
Single-stage EOFC device structures and modulation results based on SOI: (**a**) PM [127]; (**b**) DDMZM [158]; and (**c**) DPMZM [159].

**Single-stage modulation:** In recent years, research on single-pass EOFCs based on the SOI platform has shifted from early demonstrations emphasizing “tunable repetition rate and integrability” toward the co-optimization of spectral flatness and driving efficiency. Starting from a single silicon phase modulator, as shown in Figure 6a, Nagarjun et al. [127] demonstrated that a traveling-wave PN phase modulator can directly convert a continuous-wave laser into a tunable EOFC, yielding approximately eight comb lines within a single device and enabling the continuous tuning of the repetition rate from 7.5 to 12.5 GHz. However, the spectral flatness achieved remained at a relatively modest level of ~20 dB. Subsequently, Figure 6b shows that Lin et al. [158] employed a CMOS-compatible DD-MZM to advance the single-pass architecture from pure-phase modulation to interferometric spectral shaping. With a 20 GHz spacing, they generated five comb lines with a flatness of ~9 dB and reported a quantitatively extracted modulation efficiency of V_π_L ≈ 2.7 V·cm. Further progress was achieved by Liu et al. [159] in 2020, who adopted a DP-MZM configuration to further reduce V_π_L to approximately 0.9 V·cm, while generating five comb lines at a 10 GHz spacing with an improved flatness better than 2.1 dB (Figure 6c).

**Figure 7:**
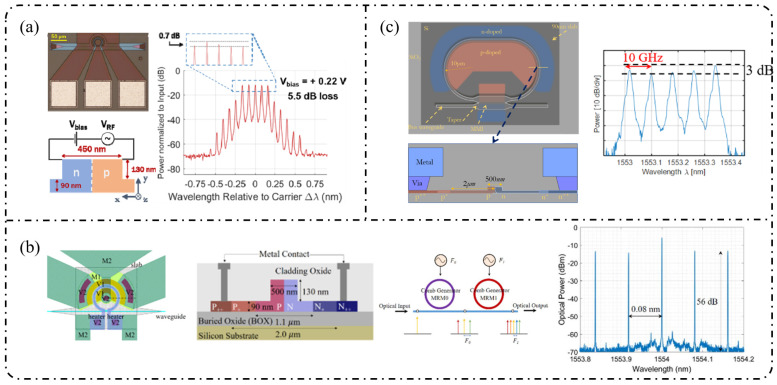
Resonant-enhanced EOFC device structures and modulation results based on SOI: (**a**) MRM [122]; (**b**) cascaded MRMs [91]; (**c**) cascaded MRMs [160].

**Resonant-enhanced modulation:** When the spectral flatness and effective modulation depth achievable with single-pass modulation are constrained by the device length and driving voltage, resonant enhancement becomes a key strategy for further improving EOFC performance. As shown in Figure 7a, Demirtzioglou et al. [122] exploited resonant field enhancement in an MRM to achieve a spectral flatness of approximately 0.7 dB with five comb lines at a 10 GHz spacing, while simultaneously reducing the effective driving requirement to V_π_∼1 V. This result clearly demonstrates that multiple intracavity round trips substantially amplify the effective modulation depth. Similarly, coupled-resonator optical waveguides (CROW) and cascaded micro-ring modulator architectures realize an effective form of “multi-stage modulation” through multi-cavity coupling. These resonant-enhanced configurations enable the generation of 5–7 comb lines with spacings ranging from 5 to 25 GHz and spectral flatness better than 3 dB under low-power operation, while naturally lending themselves to on-chip wavelength-division-multiplexed (WDM) transmitter architectures in Figure 7b [160].

**Cascaded modulation:** As single-pass silicon modulators gradually approach their intrinsic limits in terms of comb line number, spectral flatness, and modulation efficiency, EOFC implementations on the SOI platform have rapidly shifted toward finite-stage cascaded modulation architectures. The core motivation lies in the fact that coherently cascading multiple modulation units in the time and frequency domains enables simultaneous enhancement of the comb bandwidth, comb-line count, and spectral flatness without a proportional increase in the driving voltage per stage, thereby allowing precise engineering of the comb spectral envelope.

**Figure 8:**
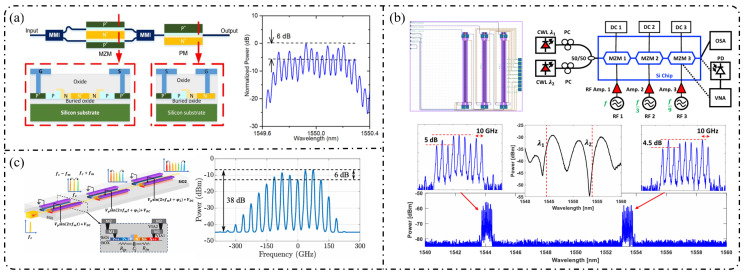
Cascaded EOFC device structures and modulation results based on SOI: (**a**) MZM + PM [161]; (**b**) cascaded 3MZMs [165]; and (**c**) cascaded 3PMs [49].

In 2018, Liu et al. [161] employed a cascaded MZM-PM configuration to generate 15 comb lines with a spacing of 5 GHz and a flatness of 6 dB and further applied the resulting EOFC to microwave synthesis in the 5–20 GHz range, clearly demonstrating the advantages of cascaded modulation in terms of spectral uniformity and scalability in Figure 8a. Subsequently, PM-MZM cascades and dual-MZM cascades were systematically developed. From a time-to-frequency (TTF) mapping perspective, Deniel et al. [162] established a unified theoretical framework for spectral flattening, showing that cascaded PM and MZM structures can generate tunable EOFCs with nine comb lines and ≤2 dB flatness over a repetition rate range of 0.1–7 GHz. Further progress was reported by Liu et al. [163] in 2020, who realized a rectangular-shaped EOFC with nine comb lines at a spacing of 5 GHz and a flatness of 1.83 dB using a two-stage cascaded MZM architecture, which directly generated 22 ps Nyquist pulses, highlighting the strength of cascaded modulation in both spectral shaping and time-domain waveform synthesis. Toward system-level integration, Wang et al. [164] and Khalil et al. [165] independently adopted two-stage and three-stage cascaded MZM configurations to generate quasi-rectangular and dual-wavelength EOFCs with nine comb lines at a 10 GHz spacing and flatness values of approximately 6–6.5 dB, thereby validating the scalability of this architecture for multi-carrier transmission and higher-order modulation formats in Figure 8b. Meanwhile, Figure 8c shows that multi-stage cascaded phase modulation with segmented driving has pushed the repetition rate to 37.5 GHz while maintaining a flatness of 6 dB over seven comb lines, representing a state-of-the-art advance for high-repetition-rate EOFCs on the SOI platform [49]. In terms of fabrication readiness, SOI benefits from highly mature CMOS-compatible processing, enabling high reproducibility and large-scale integration. However, plasma-dispersion-based modulation introduces additional optical loss and limits modulation efficiency, which constrains the achievable EOFC performance [166].

**Table 2:** Research results of different schemes based on SOI.

Scheme	Number of OFC Lines	Spacing [GHz]	Flatness [dB]	V_Π_ L [V cm]	Insert Loss/On-Chip Loss [dB]	Phase Noise [dBc/Hz]	Year
PM	8 (37)	7.5–12.5	20 (5)	N.A.	33/28	N.A.	2018 [127]
DDMZM	5	20	≈9	2.7	22.5/6.5	N.A.	2018 [158]
MRM	5	10	≈0.7	1 V (V_Π_)	5.5/N.A	N.A.	2018 [122]
Cascaded MRMs	5	10	<10	N.A.	13/2.5	N.A.	2018 [91]
Cascaded MRMs	5 (7)	10 (5)	≈4	1.6	N.A.	N.A.	2020 [160]
DP-MZM	5	10	<2	0.9	14.1/20.1	N.A.	2020 [159]
MZM + PM	15	5	<6	0.9	N.A.	N.A.	2018 [161]
PM + MZM/DDMZM	9	0.1–7	≤2	N.A.	N.A.	N.A.	2021 [162]
Cascaded MZMs	9	5	<2	0.9	20/14	−120 dBc/Hz @ 1 kHz offset	2020 [163]
Cascaded MZMs	9	5/7.5/10	<3.8/4.7/6.5	4.2	18/24	N.A.	2019 [164]
Cascaded 3 × MZMs	9	10	4.5–5	N.A.	40 (total)/N.A.	N.A.	2024 [165]
Cascaded 3 × PMs	7	37.5	≤6	2.7 × 3	25/11	N.A.	2025 [49]

### 5.2. EOFC on the InPOI

Because of its direct bandgap, the monolithic integration capability of lasers and modulators, and high carrier mobility, the InPOI platform has long been regarded as one of the most promising solutions for system-level EOFC integration. From a physical implementation perspective, recent EOFC developments on the InPOI platform have primarily followed three complementary architectural routes: single-stage modulation, aimed at compact and efficient multicarrier light sources; finite-stage cascaded modulation, intended to increase the number of comb lines and enhance spectral shaping capability; and resonant-enhanced modulation, which exploits active or passive feedback to realize effective multistage modulation and high-density spectral generation. To clarify the practical implementation landscape of these architectural paradigms, representative InP-based EOFC demonstrations are summarized in Table 3.

**Figure 9:**
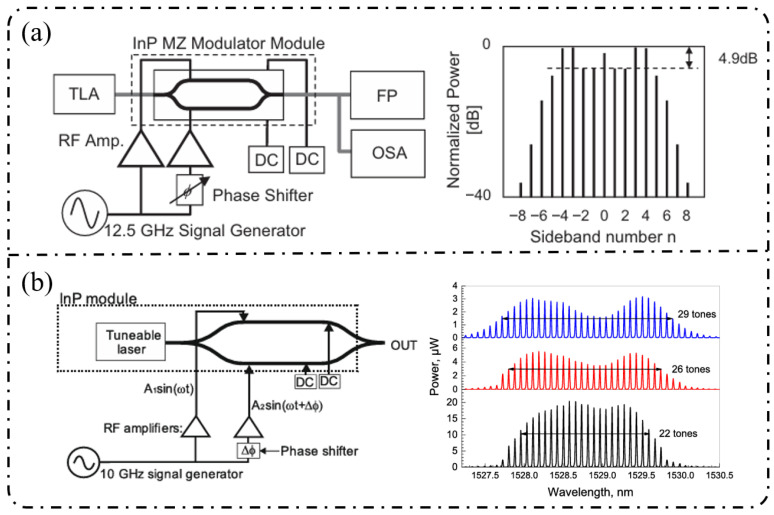
Single-stage EOFC device structures and modulation results based on InPOI: (**a**) MZM [167]; (**b**) MZM [168].

**Single-stage modulation:** In 2013, Figure 9a shows that Yamamoto et al. [167] demonstrated direct frequency comb generation using a push–pull InP MZM via sideband generation. Under 12.5 GHz RF driving, nine comb lines were obtained with a spectral ripple of 4.9 dB, and stable operation across the entire C-band (1525–1565 nm) was achieved through DC-bias control, verifying the feasibility of low-voltage, multi-carrier generation using InP MZMs. Subsequently, as shown in Figure 9b, Slavík et al. [168] further showed that a single push–pull dual-electrode InP MZM can deliver broadband EOFC output under strong RF driving, generating 29 comb lines at 10 GHz spacing and five comb lines at 20 GHz spacing, while maintaining a flatness of approximately 3 dB. In addition, the flexible control of the frequency-multiplied comb spacing was demonstrated by adjusting the relative RF phase between the two interferometer arms.

With respect to spectral-flattening mechanisms, Yokota and Yasaka systematically revealed the critical role of mixed-phase and absorption (amplitude) modulation inherent to InP MZMs in comb-spectrum shaping. Under optimized driving conditions, they achieved a quasi-rectangular EOFC comprising nine comb lines with a 12.5 GHz spacing and a flatness better than 0.8 dB, and further proposed operational strategies for additional flatness improvement, highlighting the possibility of exploiting intrinsic electro-optic nonlinearities for effective spectral equalization [169]. More recent studies have sought to push the performance limits of single-stage modulation through device-structure and process innovations. For example, slot-type EO-polymer-filled phase modulators were introduced on an InP-membrane-on-Si platform, achieving a V_π_L of approximately 0.45 V cm together with an effective modulation bandwidth of 10.5 GHz [170]. Meanwhile, Gupta et al. integrated a high-performance MZM based on multiple-quantum-well (MQW) structures and traveling-wave electrodes on the InPOI platform, realizing an electro-optic bandwidth of 32 GHz and a V_π_L of 0.7 V·cm, while supporting large-signal modulation rates up to 80 Gb/s [171].

**Figure 10:**
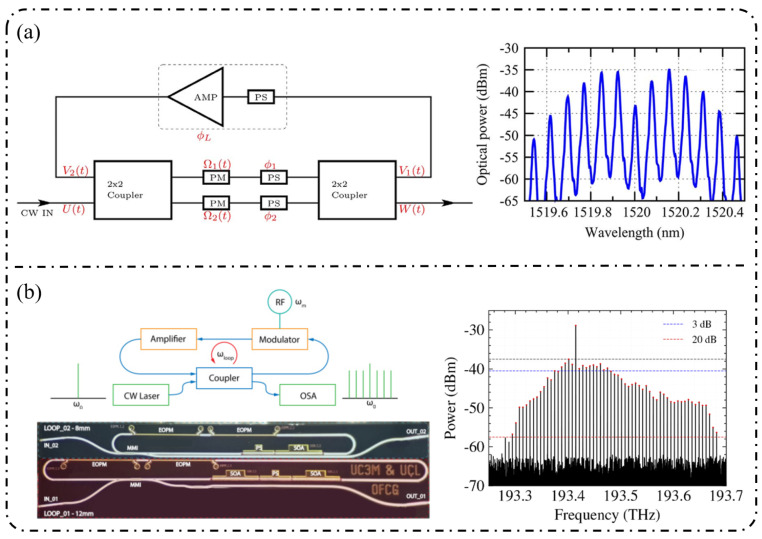
Resonant-enhanced EOFC device structures and modulation results based on InPOI: (**a**) LOOP [172]; (**b**) LOOP [173].

**Resonant-enhanced modulation:** The InPOI platform enables the monolithic integration of phase modulators and semiconductor optical amplifiers (SOAs) within feedback loops, making it inherently well-suited for resonant EOFC architectures that exploit field enhancement and multiple round-trip modulation. Dupuis et al. [172] embedded a dual-phase-modulation MZI into an optically amplified feedback loop incorporating an SOA, allowing the optical field to accumulate phase modulation over successive round trips in Figure 10a. This approach effectively realizes multistage modulation using a single modulator, generating six comb lines with continuous tunability over a wavelength span of approximately 80 nm. In 2022, as depicted in Figure 10b, Tough et al. [173] combined multistage phase modulators with a short InP waveguide feedback loop and an SOA, enabling the optical field to simultaneously accumulate modulation through both physical cascading and temporal recirculation. This resonant-enhanced configuration generated 57–59 EOFC lines (within a 20 dB spectral envelope) at repetition rates of 6.7–10 GHz, while maintaining a low synthesized phase noise of −105 dBc/Hz at a 100 kHz offset. These results highlight the unique capability of the InP-based resonant EOFCs to achieve ultrahigh comb-line counts and low phase noise through integrated amplification and feedback.

**Figure 11:**
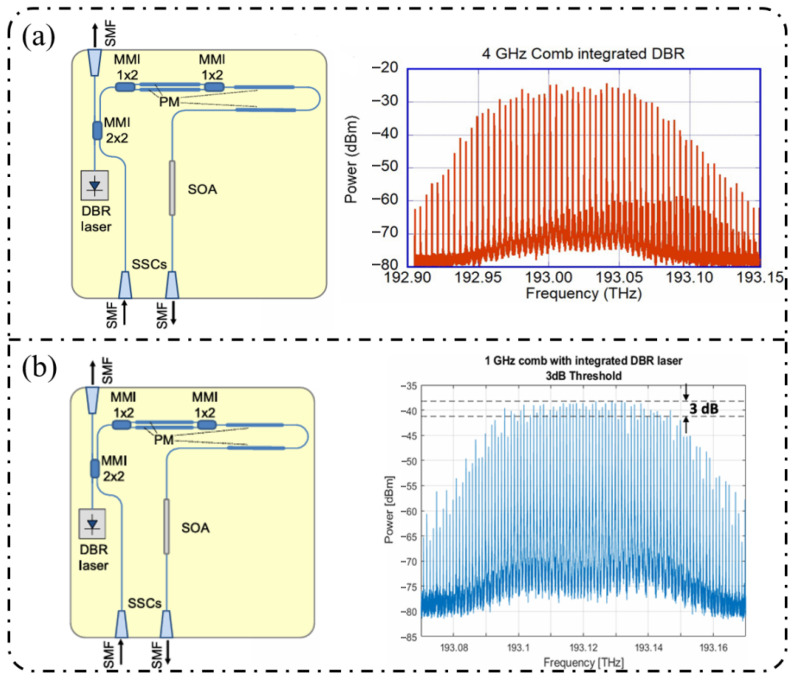
Cascaded EOFC device structures and modulation results based on InPOI: (**a**) PMs + MZM [174]; (**b**) PMs + DDMZM [175].

**Cascaded modulation:** In 2018, Andriolli et al. [174] demonstrated a fully integrated InP chip with a footprint of 4.5 × 2.5 mm^2^, incorporating a distributed Bragg reflector (DBR) laser, an MZM, two phase modulators, and an SOA in Figure 11a. This system generated up to 28 comb lines within a 5 dB power envelope at repetition rates of 4–5 GHz and could be further extended to operation at 10 GHz. Building on this architecture, Bontempi et al. [175] introduced frequency-asymmetric driving into a cascaded “dual-drive MZM + two-stage PM” configuration, where the two MZM arms were driven at frequencies f and 3f, respectively. As shown in Figure 11b, this approach enabled an on-chip comb-line multiplication mechanism, yielding 55 comb lines with a flatness of 3 dB at a fundamental repetition rate of 1 GHz and significantly improved the spectral utilization efficiency per modulation stage.

InP-based platforms exhibit strong fabrication readiness for active photonic integration due to mature epitaxial growth and device processing technologies. However, higher propagation loss, thermal sensitivity, and fabrication complexity remain key challenges for achieving low-noise and broadband EOFC operation [176,177].

### 5.3. EOFC on the LNOI

Benefiting from the synergistic advantages of the strong Pockels effect, ultrahigh refractive-index contrast, and low waveguide loss, the LNOI platform has established a technological pathway for EOFCs that is distinctly different from those of SOI and InPOI. In this platform, the modulation performance is no longer primarily constrained by carrier dynamics or absorption loss but is instead governed by the electro-optic field overlap efficiency and microwave–optical velocity matching. Consequently, EOFC implementations on LNOI generally evolve along three complementary architectures: single-stage phase or interferometric modulators to achieve large modulation indices and ultrabroadband responses, cascaded modulation structures to engineer the comb spectral envelope, and resonant or feedback-enhanced architectures that further reduce driving power while extending the number of usable comb lines. Representative EOFC implementations on the LNOI platform aligned with these architectural paradigms are summarized in Table 4, emphasizing their ultrahigh electro-optic efficiency, broadband modulation capability, and reduced driving power requirements.

**Figure 12:**
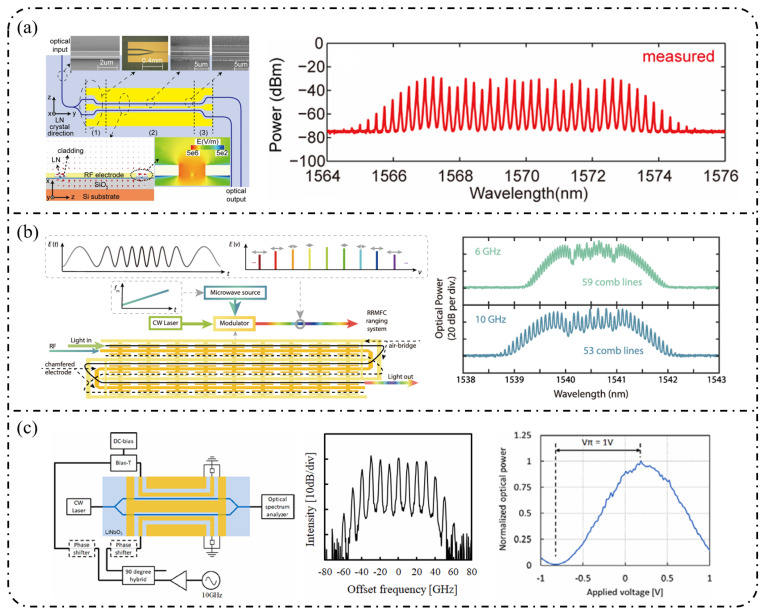
Single-stage EOFC device structures and modulation results based on LNOI: (**a**) PM [178]; (**b**) PM [179]; and (**c**) DDMZM [180].

**Single-stage modulation:** One of the most representative advantages of the LNOI platform is its ability to generate high-order EOFCs using a single modulation stage. In 2019, Ren et al. [178] employed a traveling-wave PM to generate an EOFC with more than 40 comb lines and an optical bandwidth of approximately 10 nm under 30 GHz driving (Figure 12a). The device exhibited an on-chip loss of ~4.9 dB and high RF power-handling capability (3.1 W), thereby simultaneously achieving a large bandwidth and high repetition rate without cascading. This single-stage strategy has been extended to system-level applications in recent years. In 2025, as illustrated in Figure 12b, Qi et al. [179] increased the effective modulation length to 5 cm in a folded-waveguide traveling-wave PM. While maintaining a single-stage architecture, they generated 59 comb lines at 6 GHz and 53 comb lines at 10 GHz, with remarkably low half-wave voltages of 1.47 V (3 GHz) and 1.85 V (9.5 GHz). Without relying on cavity enhancement or multistage cascading, this work achieved one of the highest comb-line counts and repetition-rate tunability reported for single-stage EOFCs on the LNOI platform. In contrast to pure phase modulation, Figure 12c shows that Takano et al. [180] demonstrated a single-stage interferometric EOFC using a DD-MZM on an LNOI. Under 10 GHz driving, nine comb lines were generated with a spectral flatness of approximately 10 dB. Benefiting from the high electro-optic efficiency of LNOI, the device exhibited an exceptionally low effective modulation efficiency of V_π_L ≈ 0.38 V·cm, highlighting the potential of amplitude–phase coupling for spectral shaping within single-stage architectures.

**Figure 13:**
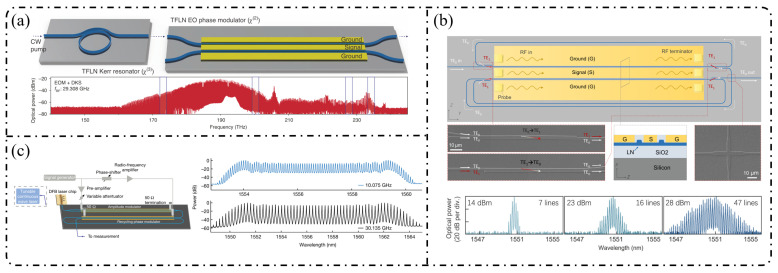
Resonant-enhanced EOFC device structures and modulation results based on LNOI: (**a**) Microresonator + PM [182]; (**b**) recycling PM (4-pass) [183]; and (**c**) MZM + Recycling PM (2-pass) [184].

**Resonant and feedback-enhanced modulation:** Another key advantage of the LNOI platform is the synergistic combination of low-loss optical waveguides and a strong Pockels effect, which enables optical-field feedback and multiple intracavity modulation events. As a result, resonant or feedback-enhanced architectures can achieve an effective modulation depth that far exceeds that of single-pass modulation. This advantage was first manifested in high-Q microcavity-based intracavity EO modulation. By 2023, the LNOI microring resonators were able to generate ≥210 comb lines at repetition rates of approximately 20 GHz [181]. Through the co-design of optical racetrack resonators and microwave resonant electrodes, EOFCs with a 25.6 GHz spacing were further extended to more than 400 comb lines while simultaneously reducing RF reflection and driving power. More recently, Song et al. [182] in 2025 combined an LNOI Kerr microresonator with external EO phase modulation to realize a hybrid Kerr–EO architecture (Figure 13a). In this scheme, a broadband optical source is first generated by dissipative Kerr solitons (DKS, with THz spacing), after which each Kerr line is split and densified via EO modulation at 29.308 GHz. This approach ultimately produced 2589 comb lines, covering 75.9 THz (588 nm) with microwave-level spacing, representing a state-of-the-art hybrid Kerr–EO frequency comb on the LNOI platform that simultaneously optimizes spectral span and repetition-rate control.

Complementary to high-Q microcavity approaches is the non-resonant feedback-enhanced pathway, in which the optical field repeatedly traverses the same modulation region within a low-loss feedback loop, thereby accumulating phase modulation while preserving broadband tunability. Figure 13b shows that using multi-loop designs with three to four round trips, the number of comb lines has been increased from 15 to 47 at a repetition rate of approximately 25 GHz, while the effective V_π_L has been reduced to 1.9 V·cm, corresponding to an approximately 15-fold reduction in RF power consumption [183]. When combined with hybrid architectures such as MZM + recycling PM in Figure 13c, this feedback-enhanced strategy enables the generation of >60–90 comb lines with repetition rates spanning 10–30 GHz and spectral flatness better than 1 dB while also supporting on-chip femtosecond pulse synthesis [184].

**Cascaded modulation:** On the LNOI platform, finite-stage cascaded modulation provides a key pathway toward an engineered optimum among a continuously tunable repetition rate, spectral flatness, and comb line number. Unlike single-stage modulation, which relies on achieving a large modulation index, or resonant enhancement, which depends on optical-field feedback, cascaded architectures combine pulse shaping via intensity modulation with spectral broadening via multistage phase modulation through time–frequency mapping. In this framework, the spectral envelope and comb line number can be controlled in a quasi-independent manner, and the front-end IM/MZM primarily determines the spectral flatness and temporal window shape, while the effective modulation depth of the subsequent PM stages governs the achievable comb-line count in an approximately linear fashion. Studies reported in 2022 demonstrated that simple cascading of an MZM (or IM) with a PM can yield approximately 13 EOFC lines over repetition rates ranging from 5 to 30 GHz while maintaining ≤1–3 dB spectral flatness, low on-chip loss (~1–2 dB), and a wide electro-optic bandwidth exceeding 60 GHz [90,114]. Subsequently, IM + PM cascades were employed to directly generate near-rectangular spectra and picosecond pulse trains, whereas IM–PM serial configurations implemented with U-shaped or compact waveguides further reduce loss and improve bias stability [185].

**Figure 14:**
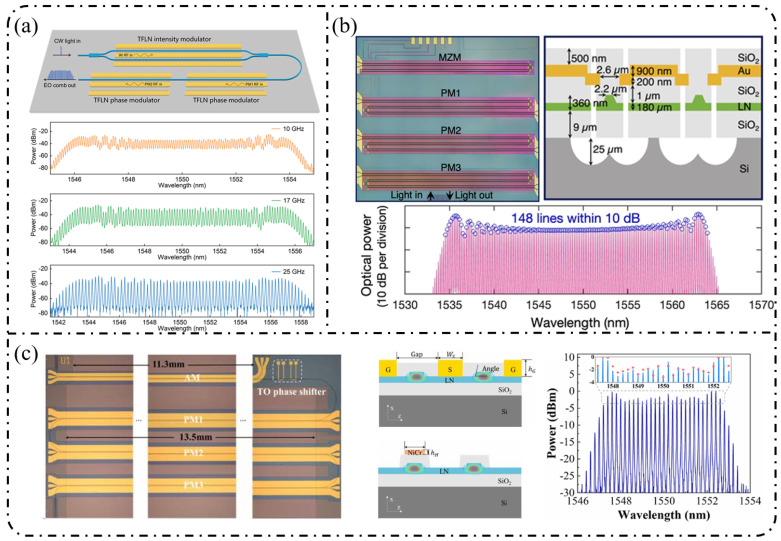
Cascaded EOFC device structures and modulation results based on LNOI: (**a**) IM + Cascaded 2PMs [186]; (**b**) MZM + Cascaded 3PMs [187]; and (**c**) AM + Cascaded 3PMs [188].

More recently, major performance advances in LNOI-cascaded EOFCs have been driven by the deep cascading of multiple-phase modulation stages. Using an IM + two-stage PM architecture, approximately 70–120 comb lines were achieved at repetition rates of 10–25 GHz while maintaining ≤3 dB flatness and a total insertion loss of ~15 dB. Figure 14a shows that these systems support continuous tuning over 5–25 GHz and offer a center-wavelength tuning range exceeding 120 nm, making them well-suited for high-order WDM systems and femtosecond pulse generation [186]. Further extending this approach, an MZM + three-stage PM cascade increased the number of usable comb lines to 148 (within a 10 dB envelope) at a 25 GHz repetition rate, spanning multiple communication bands, including S, C, L, and U, in Figure 14b. This result demonstrates that high-density, engineerable EOFCs can be realized through accumulated low-V_π_ phase modulation without relying on cavity enhancement [187].

Meanwhile, AM + three-stage PM configurations and mid-infrared cascaded LN modulators have generated flat EOFCs with ≥27 comb lines at 25 GHz spacing and a >60 nm spectral span at ~100 GHz spacing in Figure 14c. These demonstrations further underscore the versatility and scalability of the cascaded LNOI architectures across different wavelength bands and application-driven scenarios [188].

From a fabrication perspective, LNOI has rapidly evolved into one of the most mature Pockels-effect-integrated photonic platforms. Mature waveguide fabrication processes include dry-etched ridge waveguides, chemo-mechanical polishing-assisted waveguides, and hybrid-loaded structures, which enable strong optical confinement and high electro-optic efficiency [189]. However, practical limitations remain. The chemical inertness of lithium niobate makes low-damage etching challenging, and sidewall roughness can introduce non-negligible propagation loss. In addition, electrode-induced absorption, microwave loss, and wafer-scale uniformity still impose constraints on large-scale EOFC integration [190,191]. These factors may affect comb power, spectral flatness, and long-term stability in practical systems.

**Table 4:** Research results of different schemes based on LNOI.

Scheme	Number of OFC Lines	Spacing [GHz]	Flatness [dB]	V_Π_ L [V cm]	Insert Loss/On-Chip Loss [dB]	Phase Noise [dBc/Hz]	Year
PM	>40	30	10	9	16.9/4.9	N.A.	2019 [178]
PM	59/53	6/10	N.A.	7.35 (3 GHz)/9.25 (9.5 GHz)	N.A.	N.A.	2025 [179]
DDMZM	9	10	10	0.38	N.A.	N.A.	2025 [180]
Microresonator	≥210	19.5	N.A.	N.A.	N.A./6 dB/facet	N.A.	2023 [181]
Microresonator	>400	25.6	N.A.	N.A.	N.A.	N.A.	2025 [192]
Microresonator+ PM	2589	29.308	N.A.	8.84	8/6 dB/facet	N.A.	2025 [182]
Recycling PM (3-pass)	15	25	N.A.	7.38 (TE_0_)/7.35 (TE_1_)/7.52 (TE_2_)	N.A.	N.A.	2022 [193]
Recycling PM (4-pass)	47	24.95	N.A.	1.9	14/4	N.A.	2023 [183]
MZM + Recycling PM (2-pass)	91/67	10.075/30.135	≤1	5 (PM)	8/3	N.A.	2022 [184]
MZM + PM	13	31	≤1.4	2.38 (MZM)/4.76 (PM)	8.9/2.1	N.A.	2022 [114]
MZM + PM (U-type)	13	5	2.4	3.06 (MZM)/4.76 (PM)	6.97/0.8	N.A.	2022 [90]
IM + PM	25	10	≤3	10.5 (PM)/2.35 (IM)	8/N.A.	N.A.	2024 [185]
IM + Cascaded 2PMs	121/101/79	10/17/25	≤3	2.2 (IM)/2.5 V (PM)	~15/N.A.	N.A.	2025 [186]
IM + Cascaded 2PMs	67	10	10	2.6	16/N.A.	–89.53 dBc/Hz @100 kHz	2025 [109]
IM + Cascaded 2PMs	27	25	N.A.	23 (PM)/11.5 (IM)	8.9/−8.1	N.A.	2025 [194]
AM + Cascaded 3PMs	27	25	≤3	2.6 (AM)/5.2 (PM)	10.2/N.A.	N.A.	2025 [188]
MZM + Cascaded 3PMs	148	25	10	7.875 (PM)	6.5/N.A.	N.A.	2025 [187]

### 5.4. EOFC on the LTOI

In contrast to LNOI, which places greater emphasis on the ultimate electro-optic efficiency and extremely large comb-line counts, thin-film lithium tantalate on insulator (LTOI) offers distinct advantages in the EOFC domain in terms of superior device stability, lower birefringence, and weaker photorefractive effects. These characteristics make LTOI particularly well-suited as an engineering-oriented electro-optic frequency comb platform for high-optical-power operation and long-term stable performance. In recent years, EOFC implementation on the LTOI platform has primarily followed two device-level pathways. The first relies on high-speed, low-V_π_L traveling-wave MZMs for single-stage modulation, enabling direct generation of high-repetition-rate EOFCs. The second pathway exploits resonant-enhanced modulation, achieved through the co-design of optical microcavities and microwave structures, which allows the realization of extremely large comb-line counts and ultrabroad spectral coverage under a relatively low microwave driving power. Representative LTOI-based EOFC demonstrations corresponding to these device-level pathways are summarized in Table 5, highlighting their modulation efficiency, achievable comb-line counts, spectral bandwidth, and long-term operational stability.

**Figure 15:**
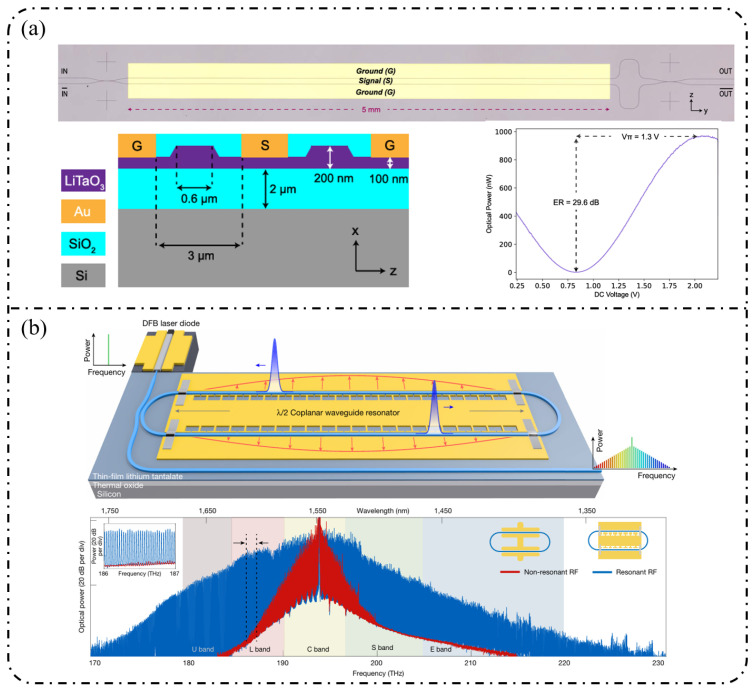
EOFC device structures and modulation results based on LTOI: (**a**) single MZM [150]; (**b**) microresonator + CPW [195].

**Single-stage modulation:** The capability of single-stage EOFC generation on the LTOI platform is primarily enabled by its high-speed, low-loss traveling-wave MZMs. In 2024, Wang et al. [149] demonstrated an LTOI-based traveling-wave MZM exhibiting a 3 dB electro-optic bandwidth of ~110 GHz and a modulation efficiency of V_π_L ≈ 2.88 V·cm. The device exhibited a typical fiber-to-chip coupling loss of ~7 dB per facet, providing a viable single-stage modulation foundation for EOFCs with repetition rates exceeding 100 GHz. Subsequently, Powell et al. [150] further improved the modulation efficiency of LTOI devices to V_π_L ≈ 0.65 V·cm, while maintaining low optical loss (5.3/4.3 dB) and minimal DC bias drift within a 20 GHz effective bandwidth in Figure 15a. These results highlight the pronounced advantage of the LTOI platform in enabling EOFC operation at a low driving voltage and high long-term stability. Heterogeneous integration approaches have also emerged. Wang et al. [92] employed SiN-LTOI hybrid waveguide structures, achieving an EO bandwidth of 67 GHz together with a modulation efficiency of V_π_L ≈ 2.75 V·cm, while preserving strong optical mode overlap. This work further demonstrates the flexibility of the LTOI platform for high-speed EOFC implementation through hybrid photonic integration.

**Resonant-enhanced modulation:** For more extreme comb-line counts and spectral spans, the advantages of the LTOI platform are fully unleashed through the synergistic enhancement of optical microcavities and microwave resonant structures. As shown in Figure 15b, Zhang et al. [195] proposed and demonstrated a triple-resonance architecture that integrates an optical microcavity with a coplanar waveguide (CPW) microwave resonator. In this configuration, the modulated optical field undergoes multiple round trips within the optical cavity, while simultaneously experiencing strong RF driving under microwave resonance. As a result, an EOFC comprising more than 2000 comb lines was generated at a repetition rate of 29.6 GHz, covering a spectral span exceeding 450 nm (>60 THz), while introducing only ~3 dB per facet of coupling loss. This work clearly demonstrates that, on the LTOI platform, the synergistic amplification of the optical quality factor (Q), microwave Q, and Pockels nonlinearity enables the realization of EOFCs with state-of-the-art comb line density and spectral coverage under relatively low RF power consumption, highlighting LTOI as a highly promising platform for ultra-broadband and high-density electro-optic frequency comb generation.

Compared with LNOI, LTOI is still an emerging platform in terms of fabrication readiness. Similar waveguide fabrication approaches, such as thin-film bonding and dry etching, can be adopted [51,196]. However, the overall process maturity, including wafer quality, etching optimization, and low-loss waveguide formation, is still under development. Propagation loss in LTOI waveguides is often influenced by etching damage and film non-uniformity. Therefore, although LTOI offers promising electro-optic and thermal properties, further process optimization is required for scalable and reproducible EOFC implementations.

From a platform perspective, no single material system simultaneously optimizes all EOFC performance metrics. LNOI provides high electro-optic efficiency and bandwidth but faces fabrication and integration challenges. SOI offers excellent scalability and fabrication maturity but is limited by plasma-dispersion-induced loss. InP platforms enable active integration but suffer from higher loss and thermal sensitivity. These platform-dependent limitations further translate into system-level constraints, including device variability, thermal stability, and integration complexity in large-scale EOFC systems. Therefore, platform selection inherently involves trade-offs between efficiency, scalability, and system integration.

## 6. Applications of EOFC

Benefiting from strictly locked repetition rates, high coherence, engineerable spectral profiles, and excellent compatibility with on-chip light sources and modulators, integrated EOFCs are rapidly evolving from mere proof-of-concept light sources to key functional building blocks in a wide range of photonic systems. In recent years, EOFCs have demonstrated pronounced advantages in precision ranging and metrology, broadband spectroscopy and sensing, and high-capacity optical communication. Their low phase noise and precisely controllable microwave spacing enable high-accuracy coherent measurements, which are well suited for parallel spectral acquisition, and their stable, highly flat frequency grids provide an ideal multiwavelength carrier foundation for dense wavelength-division multiplexing (WDM) and coherent modulation formats. In the following sections, representative system-level demonstrations and performance advantages of EOFCs across these three application domains are reviewed.

### 6.1. Precision Distance Measurement

The distinctive advantages of integrated electro-optic frequency combs (EOFCs) in precision ranging and metrology fundamentally arise from a combination of microwave-referenced comb spacing, highly coherent multicarrier operation, and engineerable spectral structures. The comb line spacing is directly defined by an RF source and can be rapidly tuned, enabling distance information to be down-converted to the RF domain for phase and frequency measurement via dual-comb or multi-heterodyne schemes or through synthetic-wavelength chains. Meanwhile, the use of a common narrow-linewidth seed laser together with electrically driven coherent locking significantly reduces the need for complex optical phase-locking infrastructure, thereby providing a system-level foundation for high refresh rates, large dynamic range, and on-chip integration.

**Figure 16:**
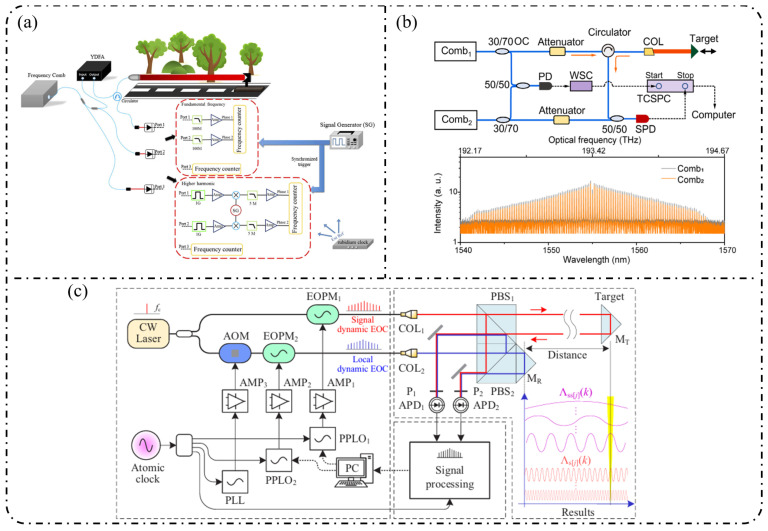
(**a**) Principle diagram of long-distance measurement using phase slope of the inter-mode beat [197]. (**b**) Experimental setup of single-photon counting laser ranging with OFCs [198]. (**c**) Schematic of multi-heterodyne interferometric absolute distance measurement based on dual dynamic EOFCs [199].

In 2020, Xu et al. [197] proposed a distance-retrieval method based on sweeping the repetition rate and extracting the distance from the phase slope of intermode beat notes, thereby breaking the reliance on ultra-stable comb spacing and complex locking loops (Figure 16a). Using this approach, they achieved indoor ranging over 65 m and outdoor ranging up to 219 m, with distance deviations controlled at the level of tens to hundreds of micrometres. This work established a methodological foundation for dynamically tunable synthetic-wavelength and phase-slope-based ranging using EOFCs. Subsequently, in 2021, Ren et al. [198] advanced a dual-EO comb ranging into a regime of extreme photon scarcity. As depicted in Figure 16b, by combining dual-comb interferometry with time-correlated single-photon counting (TCSPC), they demonstrated micrometer-level precision over a distance of 15.6 m using a 25 GHz EO comb at an optical power as low as 2.8 pW. This result highlights the feasibility of EOFC-based metrology in weak-return, low-illumination remote sensing and imaging scenarios. In 2023, Xie et al. [199] further elevated the EOFC-based range toward a traceable, scalable, and real-time metrology system (Figure 16c). Using a “dual dynamic EO comb + multi-heterodyne” architecture, they constructed a variable synthetic-wavelength chain spanning from millimeter to kilometer scales through synchronized fast frequency hopping. By implementing parallel phase demodulation on an FPGA, the real-time performance was significantly improved. Within a 45 m measurement range, their system achieved agreement with a He–Ne interferometer at the level of 8.6 µm, with a standard deviation of 0.8 µm and a resolution better than 2 µm, marking a transition of EOFC ranging from proof-of-concept demonstrations to practical, high-performance metrological systems.

**Figure 17:**
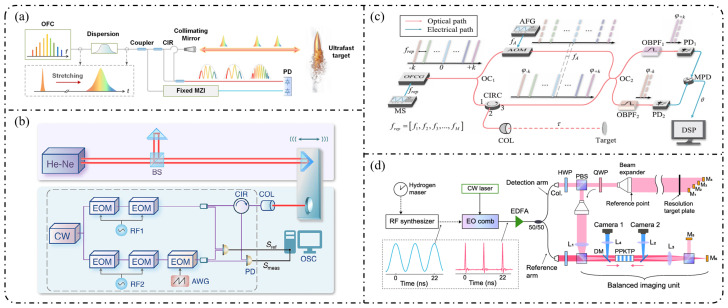
(**a**) Principle diagram of distance measurement based on OFC time-stretch resampling [200]. (**b**) Experimental setup of the dual comb ranging system [201]. (**c**) Schematic of the high-precision optical transfer delay measurement system [203]. (**d**) Experimental setup of time-domain stereoscopic imaging [202].

As of 2025, research efforts have increasingly converged toward the parallel advancement of ultrahigh speed, system integration, and multi-scenario metrological capability. On the one hand, targeting ultrafast absolute ranging, Jia et al. [200] proposed a nanosecond-scale ranging scheme based on frequency-comb time stretching and equidistant resampling, achieving a single-shot measurement cycle as short as 5 ns in Figure 17a. In experiments involving 10 m distances, non-line-of-sight (NLOS) targets, and kilohertz-vibration objects, the system achieved sub-micrometer accuracy after 1 ms averaging. By introducing an auxiliary MZI for resampling, higher-order dispersion-induced nonlinearities were directly compensated for, significantly simplifying both the system architecture and signal processing. However, for on-chip and system-level integration, Guo et al. [201] replaced conventional acousto-optic modulators (AOMs) with serrodyne-modulated electro-optic modulators (EOMs) to realize frequency shifting and remove multi-heterodyne degeneracy while simultaneously preserving coherence and integrability in Figure 17b. Their system achieved an Allan deviation below 0.1 nm at 1 ms integration, enabled tracking of vibrations up to 100 kHz, and significantly reduced RF power consumption while enhancing tuning flexibility.

During the same period, two notable extensions broadened the EOFC metrology landscape. First, repetition-rate-modulated frequency comb ranging (RRMFC) based on lithium niobate EO combs achieved a 12 GHz repetition-rate tuning range within 4 µs, pushing the single-channel absolute ranging acquisition rate to 1.79 GHz [179]. This development directly addresses the long-standing trade-off between the acquisition rate and the unambiguous range in high-speed LiDAR systems. Second, EOFC-based ranging has expanded from point measurements to parallel three-dimensional metrology. Figure 17d shows that by combining time-domain stereoscopy (TDS) with femtosecond EO-comb synthesis and nonlinear sampling, sub-100 nm depth precision was achieved over meter-scale ranges, while supporting millisecond-scale displacement and velocity measurements, together with massively parallel pixel readout [202]. This work substantially extends the applicability of EOFCs to surface metrology and precision manufacturing.

In addition, EOFCs have been applied to high-precision delay metrology in fiber links and optical networks. As shown in Figure 17c, comb-assisted phase-derived ranging demonstrated a timing accuracy of ±10.5 fs over 50 km fiber links, along with a free-space distance measurement resolution of 2.8 µm, underscoring the engineering value of a unified distance-delay-frequency metrology framework [203]. In the same year, unambiguous microwave-frequency measurement using triple EO combs was demonstrated, offering customizable, disturbance-resilient RF metrology over 0–30 GHz, with dynamic tracking capability and 1 MHz resolution. This approach provides integrated metrological support for RF references and link characterization, which are critical for EOFC-based ranging systems [204].

### 6.2. Optical Communications

The application potential of EOFCs in optical communication mainly arises from three key aspects. First, EOFCs inherently provide an array of equally spaced, mutually coherent multi-wavelength carriers, which can replace large banks of independent lasers in WDM systems and substantially reduce the grid management complexity caused by frequency drift. Second, the strong correlation between the relative frequency and phase noise among comb lines is highly advantageous for superchannel transmission and multichannel joint digital signal processing (DSP). Third, the electrically programmable and rapidly tunable repetition rate and center frequency of EOFCs facilitate seamless co-integration with modulators, filters, and routing elements on platforms such as silicon photonics and thin-film lithium niobate, enabling on-chip, reconfigurable communication light sources, and frequency-domain processing chains [34,151].

**Figure 18:**
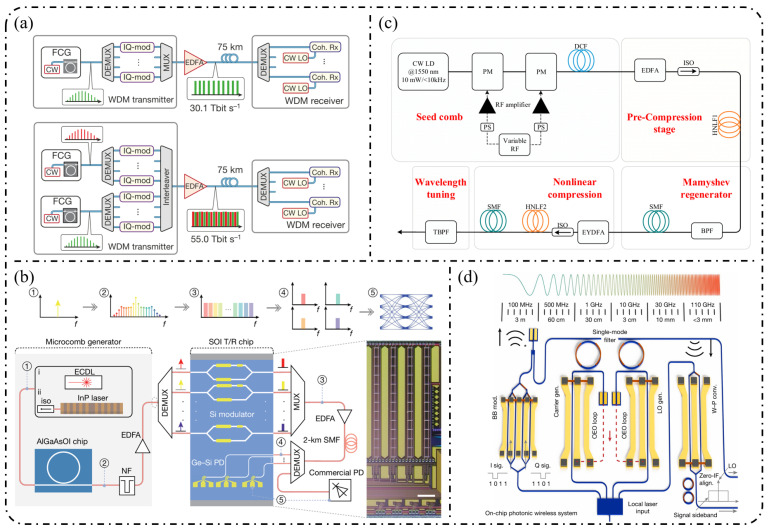
(**a**) Principle of data transmission using a single DKS comb generator as the optical source at the transmitter [205]. (**b**) Schematic of the microcomb-based data transmission set-up [206]. (**c**) The diagram of the experimental setup for the tunable electro-optic frequency comb [207]. (**d**) Schematic of the thin-film lithium niobate photonic wireless solution for ultrabroadband carrier and local oscillator generation, signal modulation and reception [208].

A representative system-level demonstration of “frequency combs as WDM carrier banks” was reported in 2017 by Marin-Palomo et al. using on-chip microresonator combs. Figure 18a shows that by employing two complementary combs—one serving as a multi-wavelength transmitter source and the other as a multi-wavelength local oscillator at the receiver—they realized coherent transmission across the C and L bands with 179 carriers and an aggregate data rate exceeding 50 Tb/s [205]. This work highlighted the scalability of frequency combs as bidirectional replacements for laser arrays at both the transmitter and receiver. In 2022, Shu et al. [206] demonstrated a communication-oriented tunable EO frequency comb based on cascaded electro-optic modulation. As shown in Figure 18b, by combining intensity and phase modulators and carefully controlling the RF drive, they generated 24 comb lines with a maximum power ripple of approximately 1.1 dB, while allowing the comb spacing to be tuned via the RF. This approach provides a flexible multicarrier light source suitable for high-capacity optical transport networks. More recently, in 2025, Zhang et al. [207] realized an all-fiber femtosecond EO frequency comb operating in the 1.5 μm band by employing cascaded phase modulation of a continuous-wave laser, followed by Mamyshev regeneration and nonlinear fiber compression. The system produced high-quality pulses with durations of approximately 470 fs over a repetition-rate range of 5.75–6.05 GHz in Figure 18c. The resulting comb simultaneously offered continuous tunability of the repetition rate, center wavelength, and spectral bandwidth across the C band (1550–1565 nm), while maintaining an RF signal-to-noise ratio of 40–55 dB, making it well suited for coherent optical communications and multi-carrier/WDM sources.

In the same year, Tao et al. [208] demonstrated a thin-film lithium niobate (TFLN) photonic-integrated platform that monolithically integrated carrier and local-oscillator generation, baseband modulation, and wireless–photonic conversion (Figure 18d). By leveraging an on-chip optoelectronic oscillator (OEO), the system provides low-phase-noise, continuously tunable RF carriers and local oscillators spanning 0.5–115 GHz. End-to-end wireless transmission was achieved across nine continuous frequency bands (5–100 GHz) with single-channel data rates up to 100 Gbps, while supporting real-time spectral reconfiguration, interference avoidance, and zero-intermediate-frequency adaptive alignment. This work demonstrates a viable on-chip photonic pathway toward 6G and full-spectrum optical–wireless converged communications.

### 6.3. Spectroscopy and Sensing

The suitability of EOFCs for spectroscopy and sensing stems from two factors. First, the comb line spacing is strictly defined by a microwave source, yielding high mutual coherence among comb lines and enabling efficient down-conversion of optical spectral information into the RF domain via multiheterodyne detection for rapid readout. Second, the line density, bandwidth, and spectral envelope of EOFCs can be engineered through the driving waveform and modulation architecture, providing flexible trade-offs among spectral resolution, refresh rate, system complexity, and integrability, features that are particularly advantageous for dual-comb spectroscopy and multichannel sensing.

**Figure 19:**
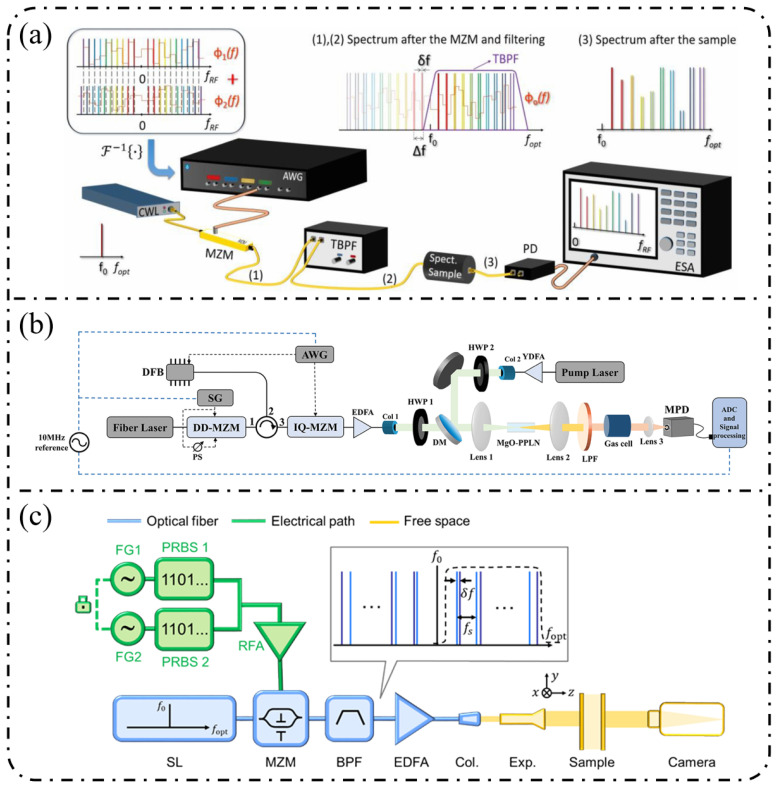
(**a**) Conceptual scheme of the common-path dual-comb interferometer, along with the signal spectrum at different points of the scheme [209]. (**b**) Conceptual scheme of the high-resolution spectrum of the mid-infrared DCS [210]. (**c**) Conceptual scheme of spatial resolution sensing achieved based on the infrared camera and dual-comb illumination device [211].

In 2020, Soriano-Amat et al. [209] significantly suppressed the uncorrelated noise arising from separate optical pathways by introducing a common-path dual-comb scheme based on a single modulator (Figure 19a). This approach enhances system stability while retaining configurational flexibility. Experimentally, they achieved approximately 3000 comb lines, an optical bandwidth of 4.5 GHz, and an optical-to-RF compression factor of 7500, enabling the measurement of narrow spectral features in the order of megahertz.

In 2024, Yuan et al. [210] leveraged digital RF synthesis combined with injection locking and difference-frequency conversion to realize a mid-infrared dual-comb system targeting the molecular “fingerprint” region. As shown in Figure 19b, an EOFC with a 13 GHz spacing was first generated in the near-infrared region; injection locking and an IQ-MZM were then employed to produce a dual comb with 50 MHz line spacing and a 13 GHz RF bandwidth. Subsequent difference-frequency generation (DFG) converted the spectrum to 3.3 μm, yielding a bandwidth of 442 GHz, a resolution of 50 MHz, and high measurement efficiency. In the same year, Escobar-Vera et al. [211] demonstrated spatially resolved dual-comb sensing by combining an infrared camera with mutually coherent dual combs generated using a single EOM. As shown in Figure 19c, by reconstructing the spectral response at each spatial pixel from a continuous image sequence, the system maintained stability over integration times exceeding 10 s, achieving 127 spectral channels, approximately 16,000 spatial positions, and a sampling rate of approximately 1 kHz for multispectral imaging and sensing. Thermally induced refractive-index changes with low etalon reflectivity are used as validation examples.

In 2025, Du et al. [212] introduced a double-sideband EO dual-comb scheme that distinguished image bands via phase biasing. Using a single commercial modulator without requiring optical filters or AOMs, the approach fully exploited the double-sideband spectral bandwidth to achieve resolved measurements over more than 200 comb lines. This work provides a more direct engineering pathway toward low-cost, low-power, on-chip dual-comb sensing units.

## 7. Summary and Prospect

This review establishes a unified analytical framework that spans the full chain of integrated EOFCs. The formation mechanisms and performance boundaries were systematically elucidated from four interconnected dimensions: electro-optic physical mechanisms, modulator architectures, key performance metrics, and material-platform dependence. Based on the four fundamental electro-optic effects, we clarify how electric-field-induced modulation of the refractive index or absorption enables controlled phase and amplitude manipulation of optical fields, and how these mechanisms are further translated into stable frequency-comb generation across different material systems. Building on this foundation, we present a unified theoretical analysis of representative modulator architectures, including PMs, MZMs, DDMZMs, and microresonator-based schemes, allowing their achievable comb bandwidth, spectral flatness, and scalability to be compared within a consistent framework.

From the perspective of system performance, the modulation depth, modulation bandwidth, optical loss, microwave–optical velocity matching, and electro-optic efficiency are identified as the core parameters that collectively determine the comb-line count, spectral flatness, output power, and phase coherence. Crucially, these factors are strongly coupled and ultimately constrained by the intrinsic physical properties of the underlying material platforms. A comparative analysis of major integrated platforms (including SOI, InPOI, and LNOI) indicates that the refractive-index contrast, electro-optic coefficients, nonlinear response, propagation loss, and fabrication compatibility jointly set the attainable performance bounds and application suitability of different EOFC implementation routes. When considered alongside representative demonstrations in precision metrology, spectroscopy, and optical communications, these insights clarify how device-level physical constraints and material platform characteristics translate into system-level EOFC capabilities.

Nevertheless, from the perspective of material platforms, the current mainstream EOFC implementations remain constrained by intrinsic physical bottlenecks. CMOS-compatible platforms such as SOI and Si_3_N_4_ benefit from low propagation loss and high refractive-index contrast, yet rely primarily on plasma-dispersion or Kerr nonlinearities for modulation, with a vanishing intrinsic second-order electro-optic response. This leads to a fundamental trade-off between modulation depth and power consumption, which is difficult to overcome. The InPOI platform enables high-speed modulation via electro-absorption effects; however, absorption loss and limited transparency windows restrict its scalability toward ultra-broadband, low-noise EOFCs. In contrast, Pockel-based platforms such as LNOI and LTOI offer stable linear electro-optic responses and low waveguide loss; however, their electro-optic coefficients are increasingly emerging as the primary bottleneck for further reducing V_π_L, expanding comb-line counts, and improving spectral flatness.

This intrinsic mismatch in material properties has directly motivated the development of heterogeneous integration and platform-convergence strategies, in which low-loss, highly mature silicon or silicon nitride photonic platforms are combined with ferroelectric thin films possessing large electro-optic coefficients, thereby simultaneously achieving a strong modulation capability and scalable on-chip system integration. More importantly, next-generation ferroelectric and relaxor-ferroelectric materials, such as BaTiO_3_, PZT, and PIN–PMN–PT, exhibit ultrahigh electro-optic coefficients (reaching several hundred to nearly one 1000 pm·V^−1^) together with broad optical transparency windows, providing a fundamentally new material pathway to overcome the existing performance limits of EOFCs in modulation efficiency, driving power consumption, and spectral broadening. Consequently, the future evolution of EOFCs will no longer rely solely on the incremental optimization of modulator architectures or microwave engineering but will increasingly depend on the integration quality, loss control, and process manufacturability of emerging electro-optic materials, thereby defining a clear research direction for next-generation EOFC platforms centered on high electro-optic coefficient ferroelectric thin films.

Further performance enhancement of EOFCs will require simultaneous improvement of modulation efficiency and bandwidth, while effectively suppressing both optical and microwave losses. Slow-light waveguides, coupled-resonator optical waveguides (CROWs), and engineered traveling-wave electrodes offer important routes for increasing the effective modulation index without sacrificing the modulation bandwidth. Simultaneously, the incorporation of ultrahigh electro-optic coefficient materials opens new degrees of freedom in EOFC design.

**Figure 20:**
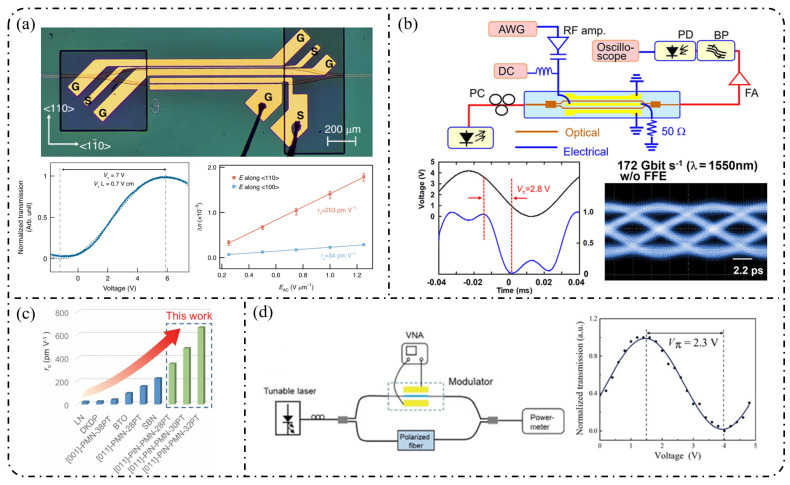
(**a**) BTO-based electro-optic modulator and its performance [213]. (**b**) PLZT-based electro-optic modulator and its performance [121]. (**c**) Comparison of EO coefficient r_c_ (at room temperature) between PIN-PMN-PT crystals and other EO crystals [156]. (**d**) PIN-PMN-PT-based electro-optic modulator and its performance [215].

Beyond lithium niobate, as shown in Figure 20, a range of emerging high-electro-optic-response material platforms has begun to enter the landscape of integrated modulators and frequency-comb systems. Strain- and domain-engineered BaTiO_3_ thin films grown on insulating substrates have demonstrated Pockel coefficients exceeding 358 pm·V^−1^, while simultaneously achieving low V_π_L values and modulation bandwidths of several tens of gigahertz at 1550 nm [213]; PZT and PLZT thin films, integrated on SiO_2_/Si platforms, offer a combination of wide optical transparency windows, large electro-optic coefficients, and hundred-of-gigahertz-class modulation capability [131,214]. Pushing the response limit even further, relaxor-ferroelectric PIN–PMN–PT single crystals, enabled by polarization and crystallographic-orientation engineering, have achieved electro-optic coefficients as high as 900 pm·V^−1^ [156]. When implemented in waveguide configurations, these materials exhibit V_π_L values far below those of commercial lithium-niobate devices, highlighting their strong potential for breaking current limits on modulation efficiency and power consumption in EOFC systems [215].

However, ultrahigh-response ferroelectric materials still face significant challenges at the device level. Their strong polarization and domain structures are essential for the electro-optic effect, yet high temperatures and residual stress during thin-film deposition, etching, and electrode integration can readily induce depolarization and domain degradation. This necessitates the development of low-temperature fabrication processes, buffer layers, and strain-engineering strategies to stabilize the polarization state. In addition, the relatively high dielectric constants of these materials substantially increase the microwave loss and impedance mismatch, rendering the design of high-speed traveling-wave electrodes and microwave–optical co-design more complex. Nevertheless, it is precisely this electro-optic response, far exceeding that of conventional materials, which provides a unique physical foundation for achieving qualitative leaps in modulation depth, driving power reduction, and attainable comb bandwidth.

It is worth emphasizing that these emerging materials are not intended to replace existing platforms, but rather to serve as critical complements in application scenarios where ultrahigh modulation efficiency and microwave–optical coupling become the dominant bottlenecks. At the device architecture level, EOFC schemes based on resonant enhancement and feedback mechanisms are expected to evolve further toward active stabilization, injection locking, or self-referenced configurations, thereby effectively translating the superior electro-optic response of advanced materials into improved comb coherence and long-term stability.

As a core interface bridging the optical and microwave domains, EOFCs uniquely combine electrically tunable repetition rates, deterministic phase coherence, and intrinsic compatibility with RF electronic systems, enabling distinctive advantages across precision-ranging, microwave–photonic radar, coherent optical communications, spectroscopy, and frequency metrology. With the continued co-evolution of microwave sources, integrated photonic chips, and digital signal processing technologies, EOFCs have been poised to develop into a general-purpose spectral engine, enabling seamless interconnection of RF, millimeter-wave, and optical frequency bands on a single chip.

Overall, EOFCs transition from laboratory prototypes to key functional modules in photonic systems. Future breakthroughs are more likely to arise from the synergistic optimization of material platforms, modulator physics, microwave–photonic engineering, and system architectures, rather than isolated advances along a single technological pathway. As these elements continue to converge, integrated electro-optic frequency combs are expected to become a foundational technology for next-generation broadband, coherent, and reconfigurable photonic systems.

## Figures and Tables

**Table 1:** Key material properties of representative integrated photonic platforms for electro-optic frequency comb generation.

Material Platform	Dominant EO Effect	Refractive Index (1550 nm)	EO Coefficient (pm·V^−1^)	Waveguide Loss (dB/cm)	Transparent Window (μm)
Si [141]	Plasma dispersion	3.49	0	3.6	1.1–8.5
Si_3_N_4_ [142,143]	Kerr	2	0	0.55	0.4–2.35
InP [144,145]	Electro-absorption	3.17	r_41_ = 1.4	0.5–4	0.9–2.75
GaAs [146,147]	Pockels	3.37	r_41_ = 1.5	1–2	0.9–12
LiNbO_3_ [148]	Pockels	2.13	r_33_ = 30.9	0.3–0.8	0.35–5
LiTaO_3_ [149,150]	Pockels	2.12	30.5	0.5	0.35–5
PZT [34,151,152]	Pockels	2.4	133	1.3–2.0	0.6–2.5
BaTiO3 [153,154]	Pockels	2.34	358	1–2	0.4–5
PIN-PMN-PT [155,156,157]	Pockels	2.47	900	N.A.	0.55–5.5

**Table 3:** Research results of different schemes based on InPOI.

Scheme	Number of OFC Lines	Spacing [GHz]	Flatness [dB]	V_Π_ L [V cm]	Insert Loss/On-Chip Loss [dB]	Phase Noise [dBc/Hz]	Year
MZM	9	12.5	4.9	2.3 V (V_Π_)	32/15	N.A.	2013 [167]
MZM	29 (5)	10 (20)	3	2.7 V (V_Π_)	6/N.A.	N.A.	2015 [168]
MZM	9 (17)	12.5	<0.8 (0.22)	2.3 V (V_Π_)	N.A.	N.A.	2016 [169]
MZM	N.A.	10.5	3	0.45	N.A./2.55	N.A.	2021 [170]
MZM	N.A.	32	3	0.7	12.5/8.5	N.A.	2023 [171]
LOOP	6	10	<5	0.19	32/14	N.A.	2012 [172]
LOOP	59	6.7	<3	0.45	>20/N.A.	−105 dBc/Hz @ 100 kHz offset	2022 [173]
PMs + MZM	28	4–5	5	1.5 (PMs)/0.5 (MZM)	5 (Fiber coupling loss)	−120 dBc/Hz @ 100 kHz offset	2018 [174]
PMs + DDMZM	55	1	3	N.A.	28 (External laser)/23 23 (On-chip laser)/18	−80~−120 dBc/Hz @ 500 Hz offset	2019 [175]

**Table 5:** Research results of different schemes based on LTOI.

Scheme	Number of OFC Lines	Spacing [GHz]	Flatness [dB]	V_Π_ L [V cm]	Insert Loss/On-Chip Loss [dB]	Phase Noise [dBc/Hz]	Year
MZM	N.A.	110	3	2.88	~7 dB/facet coupling loss	N.A.	2024 [149]
MZM	N.A.	20	3	0.65	5.3/4.3	N.A.	2025 [150]
MZM	N.A.	67	3	2.75	<1.7	N.A.	2025 [92]
Microresonator + CPW	>2000	29.6	N.A.	N.A.	3 dB/facet (coupling loss)	N.A.	2025 [195]

## Data Availability

No new data were created or analyzed in this study. Data sharing is not applicable to this article.

## References

[1] Diddams S.A., Vahala K., Udem T. (2020). Optical frequency combs: Coherently uniting the electromagnetic spectrum. Science.

[2] Fortier T., Baumann E. (2019). 20 years of developments in optical frequency comb technology and applications. Commun. Phys..

[3] Maleki L. (2011). The optoelectronic oscillator. Nat. Photonics.

[4] Gaeta A.L., Lipson M., Kippenberg T.J. (2019). Photonic-chip-based frequency combs. Nat. Photonics.

[5] Hargrove L.E., Fork R.L., Pollack M.A. (1964). Locking of He–Ne laser modes induced by synchronous intracavity modulation. Appl. Phys. Lett..

[6] Hall J.L. (2006). Nobel Lecture: Defining and measuring optical frequencies. Rev. Mod. Phys..

[7] Hänsch T.W. (2006). Nobel Lecture: Passion for precision. Rev. Mod. Phys..

[8] Obrzud E., Rainer M., Harutyunyan A., Anderson M.H., Liu J., Geiselmann M., Chazelas B., Kundermann S., Lecomte S., Cecconi M. (2018). A microphotonic astrocomb. Nat. Photonics.

[9] Papp S.B., Beha K., Del’Haye P., Quinlan F., Lee H., Vahala K.J., Diddams S.A. (2014). Microresonator frequency comb optical clock. Optica.

[10] Minoshima K., Matsumoto H. (2000). High-accuracy measurement of 240-m distance in an optical tunnel by use of a compact femtosecond laser. Appl. Opt..

[11] Riemensberger J., Lukashchuk A., Karpov M., Weng W., Lucas E., Liu J., Kippenberg T.J. (2020). Massively parallel coherent laser ranging using a soliton microcomb. Nature.

[12] Suh M.-G., Vahala K.J. (2018). Soliton microcomb range measurement. Science.

[13] Lundberg L., Karlsson M., Lorences-Riesgo A., Mazur M., Torres-Company V., Schröder J., Andrekson P.A. (2018). Frequency Comb-Based WDM Transmission Systems Enabling Joint Signal Processing. Appl. Sci..

[14] Pfeifle J., Brasch V., Lauermann M., Yu Y., Wegner D., Herr T., Hartinger K., Schindler P., Li J., Hillerkuss D. (2014). Coherent terabit communications with microresonator Kerr frequency combs. Nat. Photonics.

[15] Corcoran B., Tan M., Xu X., Boes A., Wu J., Nguyen T.G., Chu S.T., Little B.E., Morandotti R., Mitchell A. (2020). Ultra-dense optical data transmission over standard fibre with a single chip source. Nat. Commun..

[16] Temprana E., Myslivets E., Kuo B.P.P., Liu L., Ataie V., Alic N., Radic S. (2015). Overcoming Kerr-induced capacity limit in optical fiber transmission. Science.

[17] Coddington I., Swann W., Newbury N. (2008). Coherent Multiheterodyne Spectroscopy Using Stabilized Optical Frequency Combs. Phys. Rev. Lett..

[18] Suh M.-G., Yang Q.-F., Yang K.Y., Yi X., Vahala K.J. (2016). Microresonator soliton dual-comb spectroscopy. Science.

[19] Yu M., Okawachi Y., Griffith A.G., Picqué N., Lipson M., Gaeta A.L. (2018). Silicon-chip-based mid-infrared dual-comb spectroscopy. Nat. Commun..

[20] Huang C.B., Jiang Z., Leaird D., Caraquitena J., Weiner A. (2008). Spectral line-by-line shaping for optical and microwave arbitrary waveform generations. Laser Photonics Rev..

[21] Torres-Company V., Weiner A.M. (2013). Optical frequency comb technology for ultra-broadband radio-frequency photonics. Laser Photonics Rev..

[22] Marpaung D., Yao J., Capmany J. (2019). Integrated microwave photonics. Nat. Photonics.

[23] Song D., Yin K., Miao R., Zhang C., Xu Z., Jiang T. (2023). Theoretical and experimental investigations of dispersion-managed, polarization-maintaining 1-GHz mode-locked fiber lasers. Opt. Express.

[24] Guo Q., Gutierrez B.K., Sekine R., Gray R.M., Williams J.A., Ledezma L., Costa L., Roy A., Zhou S., Liu M. (2023). Ultrafast mode-locked laser in nanophotonic lithium niobate. Science.

[25] Carlson D.R., Hickstein D.D., Zhang W., Metcalf A.J., Quinlan F., Diddams S.A., Papp S.B. (2018). Ultrafast electro-optic light with subcycle control. Science.

[26] Chang L., Liu S., Bowers J.E. (2022). Integrated optical frequency comb technologies. Nat. Photonics.

[27] Herr T., Brasch V., Jost J.D., Wang C.Y., Kondratiev N.M., Gorodetsky M.L., Kippenberg T.J. (2013). Temporal solitons in optical microresonators. Nat. Photonics.

[28] Yu S.-P., Lucas E., Zang J., Papp S.B. (2022). A continuum of bright and dark-pulse states in a photonic-crystal resonator. Nat. Commun..

[29] Coillet A., Zhang S., Del’Haye P. (2022). Kerr frequency combs: A million ways to fit light pulses into tiny rings. Photoniques.

[30] Chembo Y.K. (2016). Kerr optical frequency combs: Theory, applications and perspectives. Nanophotonics.

[31] Zhang M., Buscaino B., Wang C., Shams-Ansari A., Reimer C., Zhu R., Kahn J.M., Lončar M. (2019). Broadband electro-optic frequency comb generation in a lithium niobate microring resonator. Nature.

[32] Zhang T., Yin K., Zhang C., Miao R., Jiang T. (2024). Integrated Electro-Optic Frequency Combs: Theory and Current Progress. Laser Photonics Rev..

[33] Ahn C., Na Y., Kim J. (2023). Dynamic absolute distance measurement with nanometer-precision and MHz acquisition rate using a frequency comb-based combined method. Opt. Lasers Eng..

[34] Hu H., Oxenløwe L.K. (2021). Chip-based optical frequency combs for high-capacity optical communications. Nanophotonics.

[35] Wu K., O’Malley N.P., Fatema S., Wang C., Girardi M., Alshaykh M.S., Ye Z., Leaird D.E., Qi M., Torres-Company V. (2025). Vernier microcombs for integrated optical atomic clocks. Nat. Photonics.

[36] Pan S., Ye X., Zhang Y., Zhang F. (2021). Microwave Photonic Array Radars. IEEE J. Microw..

[37] Picqué N., Hänsch T.W. (2019). Frequency comb spectroscopy. Nat. Photonics.

[38] Del’Haye P., Schliesser A., Arcizet O., Wilken T., Holzwarth R., Kippenberg T.J. (2007). Optical frequency comb generation from a monolithic microresonator. Nature.

[39] Kim J., Song Y. (2016). Ultralow-noise mode-locked fiber lasers and frequency combs: Principles, status, and applications. Adv. Opt. Photonics.

[40] Liu L., Zhang X., Xu T., Dai Z., Liu T. (2017). Simple optical frequency comb generation using a passively mode-locked quantum dot laser. Opt. Commun..

[41] Tian H., Song Y., Hu M. (2021). Noise Measurement and Reduction in Mode-Locked Lasers: Fundamentals for Low-Noise Optical Frequency Combs. Appl. Sci..

[42] Jeong H., Kim D.W., Kim H., Cha M., Moon H.S. (2024). Active offset-frequency control of optical frequency comb via sum-frequency mixing of passively mode-locked laser and continuous-wave laser. Sci. Rep..

[43] Savchenkov A.A., Matsko A.B., Liang W., Ilchenko V.S., Seidel D., Maleki L. (2011). Kerr combs with selectable central frequency. Nat. Photonics.

[44] Herr T., Hartinger K., Riemensberger J., Wang C.Y., Gavartin E., Holzwarth R., Gorodetsky M.L., Kippenberg T.J. (2012). Universal formation dynamics and noise of Kerr-frequency combs in microresonators. Nat. Photonics.

[45] Metcalf A.J., Torres-Company V., Leaird D.E., Weiner A.M. (2013). High-Power Broadly Tunable Electrooptic Frequency Comb Generator. IEEE J. Sel. Top. Quantum Electron..

[46] Wu R., Torres-Company V., Leaird D.E., Weiner A.M. (2013). Supercontinuum-based 10-GHz flat-topped optical frequency comb generation. Opt. Express.

[47] Wang C., Sun Y., Zhao Q., Yang C., Zeng C., Yang Z., Xu S. (2025). Generation and Applications of an Ultrafine and Ultraflat Electro-Optic Frequency Comb. J. Light. Technol..

[48] Wang C., Sun Y., Zhao Q., Yang C., Zeng C., Feng Z., Zhang Y., Li L., Zhou K., Wei X. (2023). Ultrafine electro-optical frequency comb based on cascade phase modulation with cyclic frequency shifting. Opt. Lett..

[49] Mohammadi A., Weckenmann E., Geravand A., Levasseur S., Rusch L.A., Shi W. (2025). High-repetition-rate electro-optic frequency combs using cascaded silicon phase modulators. Opt. Express.

[50] Zhang Y., Wu J., Jia L., Qu Y., Yang Y., Jia B., Moss D.J. (2023). Graphene Oxide for Nonlinear Integrated Photonics. Laser Photonics Rev..

[51] Li Y., Sun M., Miao T., Chen J. (2024). Towards High-Performance Pockels Effect-Based Modulators: Review and Projections. Micromachines.

[52] Zhou Z., Chao M., Su X., Fu S., Liu R., Li Z., Bo S., Chen Z., Wu Z., Han X. (2023). Silicon-Organic Hybrid Electro-Optic Modulator and Microwave Photonics Signal Processing Applications. Micromachines.

[53] Zhang W., Yao J. (2018). On-chip silicon photonic integrated frequency-tunable bandpass microwave photonic filter. Opt. Lett..

[54] Heidari E., Dalir H., Koushyar F.M., Nouri B.M., Patil C., Miscuglio M., Akinwande D., Sorger V.J. (2022). Integrated ultra-high-performance graphene optical modulator. Nanophotonics.

[55] Murai T., Kou R., Cong G., Imai M., Takabayashi K., Yamada K. (2025). 1.1-cm-long thin-film lithium niobate Mach-Zehnder modulator with low driving voltage integrated by micro-transfer printing. Opt. Express.

[56] Shen J., Zhang Y., Feng C., Xu Z., Zhang L., Su Y. (2023). Hybrid lithium tantalite-silicon integrated photonics platform for electro-optic modulation. Opt. Lett..

[57] Yu M., Wang C., Zhang M., Loncar M. (2019). Chip-Based Lithium-Niobate Frequency Combs. IEEE Photonics Technol. Lett..

[58] Ying P., Tan H., Zhang J., He M., Xu M., Liu X., Ge R., Zhu Y., Liu C., Cai X. (2021). Low-loss edge-coupling thin-film lithium niobate modulator with an efficient phase shifter. Opt. Lett..

[59] Zhang Y., Li H., Ding T., Huang Y., Liang L., Sun X., Tang Y., Wang J., Liu S., Zheng Y. (2023). Scalable, fiber-compatible lithium-niobate-on-insulator micro-waveguides for efficient nonlinear photonics. Optica.

[60] Wang Y., Jiao Y., Williams K. (2024). Scaling photonic integrated circuits with InP technology: A perspective. APL Photonics.

[61] Kashi A.A., van der Tol J.J.G.M., Lebby M.S., Zhang X., Williams K., Jiao Y. (2024). Ring-Assisted Mach-Zehnder Modulator on the InP Membrane on Silicon Platform. J. Light. Technol..

[62] Jacques M., Samani A., Patel D., El-Fiky E., Morsy-Osman M., Hoang T., Saber M.G., Xu L., Sonkoly J., Ayliffe M. (2018). Modulator material impact on chirp, DSP, and performance in coherent digital links: Comparison of the lithium niobate, indium phosphide, and silicon platforms. Opt. Express.

[63] Alexander K., George J.P., Verbist J., Neyts K., Kuyken B., Van Thourhout D., Beeckman J. (2018). Nanophotonic Pockels modulators on a silicon nitride platform. Nat. Commun..

[64] Fan G., Orobtchouk R., Han B., Li Y., Hu C., Lei L., Li H., Xu L., Wang Q. (2016). Optical Waveguides on Three Material Platforms of Silicon-on-Insulator, Amorphous Silicon and Silicon Nitride. IEEE J. Sel. Top. Quantum Electron..

[65] Reed G.T., Mashanovich G., Gardes F.Y., Thomson D.J. (2010). Silicon optical modulators. Nat. Photonics.

[66] Soref R., Bennett B. (1987). Electrooptical effects in silicon. IEEE J. Quantum Electron..

[67] Zhu G., Ji X., Zhang Z., Yan X., Yang Y., Qin F., Li X., Wu J., Sun X., Yang J. (2023). Electrically pumped optomechanical beam GaN-LED accelerometer based on the quantum-confined Stark effect. Photonics Res..

[68] Yang S., Sun X., Zhou F., Kang J., Li M., Liu X., Yan H., Luo X., Pei J., Song H. (2025). Giant built-in electric field enabled quantum-confined Stark effects. Adv. Photonics.

[69] Liu K., Ye C.R., Khan S., Sorger V.J. (2015). Review and perspective on ultrafast wavelength-size electro-optic modulators. Laser Photonics Rev..

[70] Liu J., Beals M., Pomerene A., Bernardis S., Sun R., Cheng J., Kimerling L.C., Michel J. (2008). Waveguide-integrated, ultralow-energy GeSi electro-absorption modulators. Nat. Photonics.

[71] Lines M.E., Glass A.M. (2001). Principles and Applications of Ferroelectrics and Related Materials.

[72] Almeida V.R., Xu Q., Lipson M. (2005). Ultrafast integrated semiconductor optical modulator based on the plasma-dispersion effect. Opt. Lett..

[73] Thomaschewski M., Bozhevolnyi S. (2022). Pockels modulation in integrated nanophotonics. Appl. Phys. Rev..

[74] Narasimhamurty T. (1981). Electro-optic effects in crystals: Pockels linear electro-optic and Kerr quadratic electro-optic effects. Photoelastic and Electro-Optic Properties of Crystals.

[75] Li L.J., Zhang W.H., Li H., Pan R. (2013). An overview of optical voltage sensor based on Pockels effect. Adv. Mater. Res..

[76] Hou S., Hu H., Liu Z., Xing W., Zhang J., Hao Y. (2024). High-speed electro-optic modulators based on thin-film lithium niobate. Nanomaterials.

[77] Lin G., Song Q. (2022). Kerr frequency comb interaction with Raman, Brillouin, and second order nonlinear effects. Laser Photonics Rev..

[78] Ojaghi S., Golmohammadi S., Soofi H. (2021). All-optical graphene-on-silicon slot waveguide modulator based on graphene’s Kerr effect. Appl. Opt..

[79] Chakraborty U., Carolan J., Clark G., Bunandar D., Gilbert G., Notaros J., Watts M.R., Englund D.R. (2020). Cryogenic operation of silicon photonic modulators based on the DC Kerr effect. Optica.

[80] Friedman A., Nejadriahi H., Sharma R., Fainman Y. (2021). Demonstration of the DC-Kerr effect in silicon-rich nitride. Opt. Lett..

[81] Kim Y., Takenaka M., Osada T., Hata M., Takagi S. (2014). Strain-induced enhancement of plasma dispersion effect and free-carrier absorption in SiGe optical modulators. Sci. Rep..

[82] Rahim A., Hermans A., Wohlfeil B., Petousi D., Kuyken B., Van Thourhout D., Baets R. (2021). Taking silicon photonics modulators to a higher performance level: State-of-the-art and a review of new technologies. Adv. Photonics.

[83] Takenaka M., Takagi S. (2012). Strain Engineering of Plasma Dispersion Effect for SiGe Optical Modulators. IEEE J. Quantum Electron..

[84] Reed G.T., Mashanovich G.Z., Gardes F.Y., Nedeljkovic M., Hu Y., Thomson D.J., Li K., Wilson P.R., Chen S.W., Hsu S.S. (2013). Recent breakthroughs in carrier depletion based silicon optical modulators. Nanophotonics.

[85] Chen L., Dong P., Chen Y.-K. (2012). Chirp and Dispersion Tolerance of a Single-Drive Push–Pull Silicon Modulator at 28 Gb/s. IEEE Photonics Technol. Lett..

[86] Ralph H. (1968). On the theory of the Franz-Keldysh effect. J. Phys. C Solid. State Phys..

[87] Shen H., Pollak F.H. (1990). Generalized Franz-Keldysh theory of electromodulation. Phys. Rev. B.

[88] Amin R., Khurgin J.B., Sorger V.J. (2018). Waveguide-based electro-absorption modulator performance: Comparative analysis. Opt. Express.

[89] Feng N.-N., Feng D., Liao S., Wang X., Dong P., Liang H., Kung C.-C., Qian W., Fong J., Shafiiha R. (2011). 30GHz Ge electro-absorption modulator integrated with 3 μm silicon-on-insulator waveguide. Opt. Express.

[90] Zhang Y., Wang X., Li Z., Lyu W., Lyu Y., Zeng C., Zhang Z., Zhang S., Zhang Y., Li H. (2022). Flat Optical Frequency Comb Generation Based on Monolithic Integrated LNOI Intensity and Phase Modulator. Photonics.

[91] Xu Y., Lin J., Dube-Demers R., LaRochelle S., Rusch L., Shi W. (2018). Integrated flexible-grid WDM transmitter using an optical frequency comb in microring modulators. Opt. Lett..

[92] Wang Y., Chen Y., Zhou W., Sun M., Wang X., Zhang X., Huang J., Han Q., Qu M., Su Y. (2025). High-bandwidth electro-optic modulator on a hybrid silicon-nitride-lithium-tantalate platform. Opt. Lett..

[93] Tough E.J. (2024). Integrated Optical Frequency Comb Generation For Photonic Terahertz Synthesis.

[94] Ullah S., Ullah R., Zhang Q., Khalid H.A., Memon K.A., Khan A., Tian F., Xiangjun X. (2020). Ultra-Wide and Flattened Optical Frequency Comb Generation Based on Cascaded Phase Modulator and LiNbO3-MZM Offering Terahertz Bandwidth. IEEE Access.

[95] Nagarjun K.P., Vikram B.S., Prakash R., Singh A., Selvaraja S.K., Supradeepa V.R. (2020). Optical frequency comb based on nonlinear spectral broadening of a phase modulated comb source driven by dual offset locked carriers. Opt. Lett..

[96] Chen C., He C., Zhu D., Guo R., Zhang F., Pan S. (2013). Generation of a flat optical frequency comb based on a cascaded polarization modulator and phase modulator. Opt. Lett..

[97] Yokota N., Abe K., Mieda S., Yasaka H. (2016). Harmonic superposition for tailored optical frequency comb generation by a Mach–Zehnder modulator. Opt. Lett..

[98] Zhang S., Wang Z., Zuo X., Ma C., Jiang Y., Yu J. (2023). Generation of a Flat Optical Frequency Comb via a Cascaded Dual-Parallel Mach–Zehnder Modulator and Phase Modulator without Using the Fundamental Tone. Photonics.

[99] Shi S., Yuan J., Huang Q., Shi C., Luo X., Lu S., Yuan P., Yu H., Yue Q. (2019). Bias Controller of Mach–Zehnder Modulator for Electro-Optic Analog-to-Digital Converter. Micromachines.

[100] Sakamoto T., Kawanishi T., Izutsu M. (2007). Asymptotic formalism for ultraflat optical frequency comb generation using a Mach-Zehnder modulator. Opt. Lett..

[101] Delmade A., Krstić M., Browning C., Crnjanski J., Gvozdić D., Barry L. (2019). Power efficient optical frequency comb generation using laser gain switching and dual-drive Mach-Zehnder modulator. Opt. Express.

[102] Li S., Cong R., He Z., Wang T., Zhang F., Pan S. (2020). Switchable microwave photonic filter using a phase modulator and a silicon-on-insulator micro-ring resonator. Chin. Opt. Lett..

[103] Wang J., Liu K., Harrington M.W., Rudy R.Q., Blumenthal D.J. (2022). Silicon nitride stress-optic microresonator modulator for optical control applications. Opt. Express.

[104] Kim Y., Jo Y., Kim M., Yu B.-M., Mai C., Lischke S., Zimmermann L., Choi W.-Y. (2019). Parametric optimization of depletion-type Si micro-ring modulator performances. Jpn. J. Appl. Phys..

[105] Shin M.J., Ban Y., Yu B.-M., Rhim J., Zimmermann L., Choi W.-Y. (2016). Parametric Characterization of Self-Heating in Depletion-Type Si Micro-Ring Modulators. IEEE J. Sel. Top. Quantum Electron..

[106] Adya U., Singhal S., Chen R., Chen I.T., Joshi S., Majumdar A., Li M., Moazeni S. (2025). Non-volatile tuning of cryogenic silicon photonic micro-ring modulators. Nat. Commun..

[107] Hagan D.E., Ye M., Wang P., Cartledge J.C., Knights A.P. (2020). High-speed performance of a TDFA-band micro-ring resonator modulator and detector. Opt. Express.

[108] Huang L., Yang C., Liang L., Qin L., Song Y., Lei Y., Jia P., Wang Y., Qiu C., Chen Y. (2024). Integrated Light Sources Based on Micro-Ring Resonators for Chip-Based LiDAR. Laser Photonics Rev..

[109] Li T., Ma X., Wang Z., Li X., Zhang X., Liu Z., Xie J., Chen J., Wu K. (2025). Tunable terahertz signal generation based on an on-chip wideband electro-optic frequency comb. Opt. Lett..

[110] Lü L., Gao Q., Yong M. (2022). Generation and coherent characteristics analysis of laser phase modulation spectrum by cascaded phase modulators. Optoelectron. Lett..

[111] Cui Y., Wang Z., Xu Y., Jiang Y., Yu J., Huang Z. (2022). Generation of Flat Optical Frequency Comb Using Cascaded PMs With Combined Harmonics. IEEE Photonics Technol. Lett..

[112] Sakamoto T., Chiba A. (2017). Multiple-frequency-spaced flat optical comb generation using a multiple-parallel phase modulator. Opt. Lett..

[113] Tran T.T., Song M., Song M., Seo D. (2019). Highly flat optical frequency comb generation based on pulse carving and sinusoidal phase modulation. Opt. Eng..

[114] Xu M., He M., Zhu Y., Yu S., Cai X. (2022). Flat Optical Frequency Comb Generator Based on Integrated Lithium Niobate Modulators. J. Light. Technol..

[115] Li C., Chen B., Ruan Z., Wu H., Zhou Y., Liu J., Chen P., Chen K., Guo C., Liu L. (2022). High modulation efficiency and large bandwidth thin-film lithium niobate modulator for visible light. Opt. Express.

[116] Luo B., He J.-R. (2022). Light bullet solutions in a dual-core waveguide amplifier with different modulated coefficients and a varying source. Indian J. Phys..

[117] Sekine N., Toprasertpong K., Takagi S., Takenaka M. (2020). Numerical analyses of optical loss and modulation bandwidth of an InP organic hybrid optical modulator. Opt. Express.

[118] Tang H., Li Q., Ho C.P., Fujikata J., Noguchi M., Takahashi S., Toprasertpong K., Takagi S., Takenaka M. (2022). Modulation bandwidth improvement of III-V/Si hybrid MOS optical modulator by reducing parasitic capacitance. Opt. Express.

[119] Yu H., Ying D., Pantouvaki M., Van Campenhout J., Absil P., Hao Y., Yang J., Jiang X. (2014). Trade-off between optical modulation amplitude and modulation bandwidth of silicon micro-ring modulators. Opt. Express.

[120] O’Sullivan D., McCarthy J., Russell E., Jia Z., Peters F.H., Kelleher B. (2025). Optical frequency comb bandwidth enhancement of a monolithically integrated gain switched device. Opt. Express.

[121] Xing Y., Wang Q., Huo L., Lou C. (2013). Frequency chirp linearization for ultraflat optical frequency comb generation based on group velocity dispersion. Opt. Lett..

[122] Demirtzioglou I., Lacava C., Bottrill K.R.H., Thomson D.J., Reed G.T., Richardson D.J., Petropoulos P. (2018). Frequency comb generation in a silicon ring resonator modulator. Opt. Express.

[123] Muhanad Fadhel M., Rashid H., Essa Hamzah A., Dzulkefly Zan M.S., Abd Aziz N., Arsad N. (2021). Flat frequency comb generation employing cascaded single-drive Mach–Zehnder modulators with a simple analogue driving signal. J. Mod. Opt..

[124] Shen J., Wu S. (2019). Ultra-flat optical frequency comb generation based on amplitude modulator and Gaussian band-stop filter. Opt. Quantum Electron..

[125] Torcheboeuf N., Buchs G., Kundermann S., Portuondo-Campa E., Bennès J., Lecomte S. (2017). Repetition rate stabilization of an optical frequency comb based on solid-state laser technology with an intra-cavity electro-optic modulator. Opt. Express.

[126] Okubo S., Onae A., Nakamura K., Udem T., Inaba H. (2018). Offset-free optical frequency comb self-referencing with an f-2f interferometer. Optica.

[127] Nagarjun K.P., Jeyaselvan V., Selvaraja S.K., Supradeepa V.R. (2018). Generation of tunable, high repetition rate optical frequency combs using on-chip silicon modulators. Opt. Express.

[128] Kang J., Feng P., Li B., Zhang C., Wei X., Lam E.Y., Tsia K.K., Wong K.K.Y. (2018). Video-rate centimeter-range optical coherence tomography based on dual optical frequency combs by electro-optic modulators. Opt. Express.

[129] Eliason T., Parker P.A., Reber M.A.R. (2024). Electro-optic frequency comb generation via cascaded modulators driven at lower frequency harmonics. Opt. Express.

[130] Parriaux A., Hammani K., Millot G. (2020). Electro-optic frequency combs. Adv. Opt. Photonics.

[131] Mao J., Uemura F., Yazdani S.A., Yin Y., Sato H., Lu G.-W., Yokoyama S. (2024). Ultra-fast perovskite electro-optic modulator and multi-band transmission up to 300 Gbit s−1. Commun. Mater..

[132] Heebøll H.R., Sekhar P., Riebesehl J., Razumov A., Heyrich M., Galili M., Da Ros F., Diddams S.A., Zibar D. (2024). Resonant EO combs: Beyond the standard phase noise model of frequency combs. Opt. Express.

[133] Razumov A., Heebøll H.R., Dummont M., Terra O., Dong B., Riebesehl J., Varming P., Pedersen J.E., Ros F.D., Bowers J.E. (2023). Subspace tracking for phase noise source separation in frequency combs. Opt. Express.

[134] Krause B.L., Lavrič A., Tebart J., Stöhr A., Preu S. (2024). Phase noise of an electro-optic terahertz comb. Opt. Express.

[135] Sharma V., Singh S., Lovkesh (2022). Design of tunable optical frequency comb generation based on electro-optic modulator. Photonic Netw. Commun..

[136] Shi Y., Yang Y.-D., Hu Z.-H., Hu B.-W., Dong Z., Xiao J.-L., Chen Y.-L., Huang Y.-Z. (2025). Optical frequency comb generation based on spectral broadening of a self-injection locked directly modulated microcavity laser. Opt. Express.

[137] Shang L., Li Y., Wu F. (2019). Optical frequency comb generation using a polarization division multiplexing Mach–Zehnder modulator. J. Opt..

[138] Shao X., Han H., Wang H., Ma J., Hu Y., Li C., Teng H., Chang G., Wang B., Wei Z. (2023). High power optical frequency comb with 10−19 frequency instability. Opt. Express.

[139] Chen J., Liu S., Wu K., Xiao X. (2017). Power efficient ultraflat optical frequency comb generation by cascading modulators. Opt. Eng..

[140] Nagarjun K.P., Raj P., Jeyaselvan V., Selvaraja S.K., Supradeepa V.R. (2020). Microwave power induced resonance shifting of silicon ring modulators for continuously tunable, bandwidth scaled frequency combs. Opt. Express.

[141] Vlasov Y., McNab S. (2004). Losses in single-mode silicon-on-insulator strip waveguides and bends. Opt. Express.

[142] Dabos G., Manolis A., Giesecke A.L., Porschatis C., Chmielak B., Wahlbrink T., Pleros N., Tsiokos D. (2017). TM grating coupler on low-loss LPCVD based Si3N4waveguide platform. Opt. Commun..

[143] Bauters J.F., Heck M.J., John D., Dai D., Tien M.C., Barton J.S., Leinse A., Heideman R.G., Blumenthal D.J., Bowers J.E. (2011). Ultra-low-loss high-aspect-ratio Si3N4 waveguides. Opt. Express.

[144] Zhang K., Böhm G., Belkin M.A. (2022). Mid-infrared microring resonators and optical waveguides on an InP platform. Appl. Phys. Lett..

[145] Smit M., Leijtens X., Ambrosius H., Bente E., van der Tol J., Smalbrugge B., de Vries T., Geluk E.-J., Bolk J., van Veldhoven R. (2014). An introduction to InP-based generic integration technology. Semicond. Sci. Technol..

[146] Chang L., Boes A., Guo X., Spencer D.T., Kennedy M.J., Peters J.D., Volet N., Chiles J., Kowligy A., Nader N. (2018). Heterogeneously Integrated GaAs Waveguides on Insulator for Efficient Frequency Conversion. Laser Photonics Rev..

[147] Berseth C.A., Wuethrich C., Reinhart F.K. (1992). The electro-optic coefficients of GaAs: Measurements at 1.32 and 1.52 μm and study of their dispersion between 0.9 and 10 μm. J. Appl. Phys..

[148] Cai L., Mahmoud A., Piazza G. (2019). Low-loss waveguides on Y-cut thin film lithium niobate: Towards acousto-optic applications. Opt. Express.

[149] Wang C., Fang D., Zhang J., Kotz A., Lihachev G., Churaev M., Li Z., Schwarzenberger A., Ou X., Koos C. (2024). Ultrabroadband thin-film lithium tantalate modulator for high-speed communications. Optica.

[150] Powell K., Renaud D., Li X., Assumpcao D., Xin C.J., Sinclair N., Lončar M. (2025). A sub-volt near-IR lithium tantalate electro-optic modulator. APL. Photonics.

[151] Torres-Company V., Schroder J., Fulop A., Mazur M., Lundberg L., Helgason O.B., Karlsson M., Andrekson P.A. (2019). Laser Frequency Combs for Coherent Optical Communications. J. Light. Technol..

[152] Teowee G., Boulton J.M., Franke E.K., Motakef S., Alexander T.P., Bukowski T.J., Uhlmann D.R. (2006). Optical waveguide losses of pzt thin films with various zr/ti stoichiometries. Integr. Ferroelectr..

[153] Zhu M., Du Z., Jing L., Yoong Tok A.I., Tong Teo E.H. (2015). Optical and electro-optic anisotropy of epitaxial PZT thin films. Appl. Phys. Lett..

[154] Petraru A., Siegert M., Schmid M., Schubert J., Buchal C. (2011). Ferroelectic BaTiO_3_ Thin Film Optical Waveguide Modulators. MRS Proc..

[155] Zhang Y., Zhuang Y., Yang L., Liu X., Hu Q., Yan H., Zhang H., Zhang Y., Zhang S., Del’Haye P. (2025). Monolithic electric field control of a grating coupler for finely tuning wavelength, efficiency, and bandwidth. Opt. Lett..

[156] Liu X., Tan P., Ma X., Wang D., Jin X., Liu Y., Xu B., Qiao L., Qiu C., Wang B. (2022). Ferroelectric crystals with giant electro-optic property enabling ultracompact Q-switches. Science.

[157] Hu Q., Zhang Y., Liao H., Liu X., Li P., Zhuang Y., Xu Z., Wei X. (2023). Fabrication of titanium in-diffusion waveguide in the PIN-PMN-PT single crystal. Ceram. Int..

[158] Lin J., Sepehrian H., Xu Y., Rusch L.A., Shi W. (2018). Frequency Comb Generation Using a CMOS Compatible SiP DD-MZM for Flexible Networks. IEEE Photonics Technol. Lett..

[159] Liu S., Wu K., Zhou L., Lu L., Zhang B., Zhou G., Chen J. (2020). Microwave Pulse Generation With a Silicon Dual-Parallel Modulator. J. Light. Technol..

[160] Mostafa Khalil R.M., Naghdi B., Samani A., Jacques M., Chen L.R., Plant D.V. (2020). Electro-Optic Frequency Comb Generation Using Cascaded Silicon Microring Modulators. Integrated Photonics Research, Silicon and Nanophotonics.

[161] Liu S.Q., Wu K., Zhou L.J., Xiao X., Zhong Y.M., Chen J.P. (2018). Optical frequency comb generation and microwave synthesis with integrated cascaded silicon modulators. Conference on Lasers and Electro-Optics.

[162] Deniel L., Weckenmann E., Pérez Galacho D., Lafforgue C., Monfray S., Alonso-Ramos C., Bramerie L., Boeuf F., Viven L., Marris-Morini D. (2021). Silicon photonics phase and intensity modulators for flat frequency comb generation. Photonics Res..

[163] Liu S., Wu K., Zhou L., Lu L., Zhang B., Zhou G., Chen J. (2020). Optical Frequency Comb and Nyquist Pulse Generation With Integrated Silicon Modulators. IEEE J. Sel. Top. Quantum Electron..

[164] Wang Z., Ma M., Sun H., Khalil M., Adams R., Yim K., Jin X., Chen L.R. (2019). Optical Frequency Comb Generation Using CMOS Compatible Cascaded Mach–Zehnder Modulators. IEEE J. Quantum Electron..

[165] Khalil M., Sun H., Papatheodorakos T., Adams R., Chen L.R. Optical Frequency Comb Generation Using Integrated Cascaded MZMs on SOI. Proceedings of the 2024 Photonics North (PN).

[166] Hu H., Yang J., Gui L., Sohler W. Lithium niobate-on-insulator (LNOI): Status and perspectives. Proceedings of the Silicon Photonics and Photonic Integrated Circuits III.

[167] Yamamoto T., Hitomi K., Kobayashi W., Yasaka H. (2013). Optical Frequency Comb Block Generation by Using Semiconductor Mach–Zehnder Modulator. IEEE Photonics Technol. Lett..

[168] Slavík R., Farwell S.G., Wale M.J., Richardson D.J. (2015). Compact Optical Comb Generator Using InP Tunable Laser and Push-Pull Modulator. IEEE Photonics Technol. Lett..

[169] Yokota N., Yasaka H. (2016). Operation Strategy of InP Mach–Zehnder Modulators for Flat Optical Frequency Comb Generation. IEEE J. Quantum Electron..

[170] Kashi A.A., Van der Tol J.J.G.M., Williams K.A., Yao W., Lebby M.S., Pecinovsky C., Jiao Y. (2021). Electro-Optic Slot Waveguide Phase Modulator on the InP Membrane on Silicon Platform. IEEE J. Quantum Electron..

[171] Gupta Y.D., Binet G., Diels W., Abdeen O., Gaertner T., Baier M., Schell M. (2023). Implementation, Modelling and Verification of High-Speed Mach-Zehnder Phase Modulators in an Open Access InP Foundry Platform. J. Light. Technol..

[172] Dupuis N., Doerr C.R., Zhang L., Chen L., Sauer N.J., Dong P., Buhl L.L., Ahn D. (2012). InP-Based Comb Generator for Optical OFDM. J. Light. Technol..

[173] Tough E.J., Fice M.J., Carpintero G., Renaud C.C., Seeds A.J., Balakier K. (2022). InP integrated optical frequency comb generator using an amplified recirculating loop. Opt. Express.

[174] Andriolli N., Cassese T., Chiesa M., de Dios C., Contestabile G. (2018). Photonic Integrated Fully Tunable Comb Generator Cascading Optical Modulators. J. Light. Technol..

[175] Bontempi F., Andriolli N., Scotti F., Chiesa M., Contestabile G. (2019). Comb Line Multiplication in an InP Integrated Photonic Circuit Based on Cascaded Modulators. IEEE J. Sel. Top. Quantum Electron..

[176] Yan Z., Han Y., Lin L., Xue Y., Ma C., Ng W.K., Wong K.S., Lau K.M. (2021). A monolithic InP/SOI platform for integrated photonics. Light Sci. Appl..

[177] Xie Z., Bo F., Lin J., Hu H., Cai X., Tian X.-H., Fang Z., Chen J., Wang M., Chen F. (2024). Recent development in integrated Lithium niobate photonics. Adv. Phys. X.

[178] Ren T., Zhang M., Wang C., Shao L., Reimer C., Zhang Y., King O., Esman R., Cullen T., Loncar M. (2019). An Integrated Low-Voltage Broadband Lithium Niobate Phase Modulator. IEEE Photonics Technol. Lett..

[179] Qi Y., Jia X., Wang J., Yang W., Miao Y., Cai X., Wu G., Li Y. (2025). 1.79-GHz acquisition rate absolute distance measurement with lithium niobate electro-optic comb. Nat. Commun..

[180] Takano S., Murakami S., Yamaguchi Y., Akahane K., Sakamoto T., Takigawa R. (2025). Lithium Niobate on Insulator Modulator in Dual-Drive Configuration for Optical Frequency Comb Generation. CLEO: Science and Innovations.

[181] Niu R., Wan S., Li W., Wang P.-Y., Sun F.-W., Bo F., Liu J., Guo G.-C., Dong C.-H. (2023). An integrated wavemeter based on fully-stabilized resonant electro-optic frequency comb. Commun. Phys..

[182] Song Y., Hu Y., Loncar M., Yang K. (2025). Hybrid Kerr-electro-optic frequency combs on thin-film lithium niobate. Light Sci. Appl..

[183] Zhang K., Sun W., Chen Y., Feng H., Zhang Y., Chen Z., Wang C. (2023). A power-efficient integrated lithium niobate electro-optic comb generator. Commun. Phys..

[184] Yu M., Barton D., Cheng R., Reimer C., Kharel P., He L., Shao L., Zhu D., Hu Y., Grant H.R. (2022). Integrated femtosecond pulse generator on thin-film lithium niobate. Nature.

[185] Wang X., Li Z., Chen J., Shang C., Zhang Z., Li H., Liu Y., Zeng C., Xia J. (2024). Integrated thin-film lithium niobate electro-optic frequency comb for picosecond optical pulse train generation. Appl. Phys. Lett..

[186] Wen H., Wang J., Zhu L., Shi L., Zhang X. (2025). Broadband and flat-top integrated electro-optic frequency combs on a thin-film lithium niobate platform. Opt. Lett..

[187] Wang J., Wang Q., Xu M., Zhu Y., Wang Y., Deng H., Cai X. (2025). Highly tunable flat-top thin-film lithium niobate electro-optic frequency comb generator with 148 comb lines. Opt. Express.

[188] Li D., Li R., Li M., Han M., Liu J., An J., Wang S., Li J. (2025). Broadband flat thin-film lithium niobate electro-optic frequency comb generator fabricated with photolithography. Opt. Express.

[189] Jia Y., Wang L., Chen F. (2021). Ion-cut lithium niobate on insulator technology: Recent advances and perspectives. Appl. Phys. Rev..

[190] Khalid M., Ferraresi S., Bellanca G., Barbiroli M., Fuschini F., Tralli V., Bertozzi D., Petruzzelli V., Calò G. (2024). LNOI wireless switches based on optical phased arrays for on-chip communication. IEEE J. Sel. Areas Commun..

[191] Hu L., Wang B., Guo Y., Du S., Chen J., Li J., Gu C., Wang L. (2022). Quasi-BIC enhanced broadband terahertz generation in all-dielectric metasurface. Adv. Opt. Mater..

[192] Chen Z., Zhang Y., Feng H., Zeng Y., Zhang K., Wang C. (2025). Microwave-resonator-enabled broadband on-chip electro-optic frequency comb generation. Photonics Res..

[193] Huang H., Han X., Balčytis A., Dubey A., Boes A., Nguyen T.G., Ren G., Tan M., Tian Y., Mitchell A. (2022). Non-resonant recirculating light phase modulator. APL Photonics.

[194] Ren D., Ming X., Ma K., Shi L., Sun Q., Wang L., Zhao W., Zhang W. (2025). Mid-infrared comb generator using cascaded high-speed lithium niobate modulators. J. Opt..

[195] Zhang J., Wang C., Denney C., Riemensberger J., Lihachev G., Hu J., Kao W., Blesin T., Kuznetsov N., Li Z. (2025). Ultrabroadband integrated electro-optic frequency comb in lithium tantalate. Nature.

[196] Zhu Y., Wan Q. (2024). Lithium niobate/lithium tantalate single-crystal thin films for post-moore era chip applications. Moore More.

[197] Xu X., Zhang Z., Zhang H., Zhao H., Xia W., He M., Li J., Zhai J., Wu H. (2020). Long distance measurement by dynamic optical frequency comb. Opt. Express.

[198] Ren X., Xu B., Fei Q., Liang Y., Ge J., Wang X., Huang K., Yan M., Zeng H. (2021). Single-Photon Counting Laser Ranging With Optical Frequency Combs. IEEE Photonics Technol. Lett..

[199] Xie J., Yan L., Chen B., Lou Y., Guo G. (2023). Multi-heterodyne interferometric absolute distance measurements based on dual dynamic electro-optic frequency combs. Opt. Express.

[200] Jia L., Zhang F., Qu X. (2025). High-precision nanosecond ranging based on optical frequency comb time-stretch resampling. Opt. Lasers Eng..

[201] Guo X., Yang X., Zhi J., Shao C., Wu H. (2025). Serrodyne-Enabled Dual Electro-Optic Comb Interferometry for High-Precision Absolute Ranging and Integration-Ready Metrology. Adv. Sci..

[202] Wang Z., Ma H., Luo J., Yan M., Huang K., Fang J., Ge J., Zeng H. (2025). High-precision time-domain stereoscopic imaging with a femtosecond electro-optic comb. Nat. Commun..

[203] Liu X., Zhao X., Wang B., Zhang W. (2025). High-Precision Optical Transfer Delay Measurement Using Optical-Comb-Assisted Phase-Derived Ranging. IEEE Photonics Technol. Lett..

[204] Yuan Z., Fan X., Sun P., Wan Y., Xu B., He Z. (2025). Non-Ambiguous Microwave Frequency Measurement Based on Triple Electro-Optic Frequency Comb. IEEE Photonics Technol. Lett..

[205] Lukens J.M., Lu H.-H., Qi B., Lougovski P., Weiner A.M., Williams B.P. (2020). All-Optical Frequency Processor for Networking Applications. J. Light. Technol..

[206] Shu H., Chang L., Tao Y., Shen B., Xie W., Jin M., Netherton A., Tao Z., Zhang X., Chen R. (2022). Microcomb-driven silicon photonic systems. Nature.

[207] Zhang A., Dai K., Huang L., Sheng L., Liu Z., Cui Y., Hao X., Zhang Y. (2025). Tunable All-Fiber Femtosecond Electro-Optic Optical Frequency Comb Operating at 1.5 μm. Photonics.

[208] Tao Z., Wang H., Feng H., Guo Y., Shen B., Sun D., Tao Y., Han C., He Y., Bowers J.E. (2025). Ultrabroadband on-chip photonics for full-spectrum wireless communications. Nature.

[209] Soriano-Amat M., Soto M.A., Duran V., Martins H.F., Martin-Lopez S., Gonzalez-Herraez M., Fernandez-Ruiz M.R. (2020). Common-Path Dual-Comb Spectroscopy Using a Single Electro-Optic Modulator. J. Light. Technol..

[210] Yuan Z., Fan X., Xu B., Zhu Y., He Z. (2024). Digitally generated high-resolution mid-infrared dual-comb spectroscopy system based on electro-optic modulation. Opt. Lett..

[211] Escobar-Vera C., Moreno-Oyervides A., Soriano-Amat M., Martin-Lopez S., Bonilla-Manrique O.E., Fernandez-Ruiz M.R., Gonzalez-Herraez M., Martin-Mateos P., Duran V. (2024). Spatially resolved dual-comb sensing with a single electro-optic modulator. Opt. Express.

[212] Du Y., Zheng Z., Zhao X. (2025). Double-Sideband Electro-Optic Dual-Comb Spectroscopy Using One Modulator. Laser Photonics Rev..

[213] Deng C., He Y., Yang W., Yu H., Hong Z., Liu H., Han H., Li W., Ma Y., Zhang Z. (2026). Self-buffered epitaxy of barium titanate on oxide insulators enables high-performance electro-optic modulators. Light Sci. Appl..

[214] Yu H., Xie Y., Li C., Wang J., Yang C., Wang L., Li K., Dai D., Li M., Qiu F. (2025). Thin-Film Lead Zirconate Titanate Nanobeam Electro-Optic Modulator. ACS Photonics.

[215] Hu Q., Zhang Y., Liao H., Liu X., Li P., Feng Y., An L., Zhuang Y., Xu Z., Wei X. (2023). Greatly enhanced electro-optic modulation efficiency in titanium in-diffusion PIN–PMN–PT waveguide. J. Adv. Ceram..

